# Multi-omics analysis reveals RNA splicing alterations and their biological and clinical implications in lung adenocarcinoma

**DOI:** 10.1038/s41392-022-01098-5

**Published:** 2022-08-22

**Authors:** Quanyou Wu, Lin Feng, Yaru Wang, Yousheng Mao, Xuebing Di, Kaitai Zhang, Shujun Cheng, Ting Xiao

**Affiliations:** 1 State Key Laboratory of Molecular Oncology, Department of Etiology and Carcinogenesis, National Cancer Center/National Clinical Research Center for Cancer/Cancer Hospital, Chinese Academy of Medical Sciences and Peking Union Medical College, Beijing, 100021 China; 2 Department of Thoracic Surgery, National Cancer Center/National Clinical Research Center for Cancer/Cancer Hospital, Chinese Academy of Medical Sciences and Peking Union Medical College, Beijing, 100021 China

**Keywords:** Lung cancer, Cancer genetics, Cancer genomics

## Abstract

Alternative RNA splicing is one of the most important mechanisms of posttranscriptional gene regulation, which contributes to protein diversity in eukaryotes. It is well known that RNA splicing dysregulation is a critical mechanism in tumor pathogenesis and the rationale for the promising splice-switching therapeutics for cancer treatment. Although we have a comprehensive understanding of DNA mutations, abnormal gene expression profiles, epigenomics, and proteomics in lung adenocarcinoma (LUAD), little is known about its aberrant alternative splicing profiles. Here, based on the multi-omics data generated from over 1000 samples, we systematically studied the RNA splicing alterations in LUAD and revealed their biological and clinical implications. We identified 3688 aberrant alternative splicing events (AASEs) in LUAD, most of which were alternative promoter and exon skip. The specific regulatory roles of RNA binding proteins, somatic mutations, and DNA methylations on AASEs were comprehensively interrogated. We dissected the functional implications of AASEs and concluded that AASEs mainly affected biological processes related to tumor proliferation and metastasis. We also found that one subtype of LUAD with a particular AASEs pattern was immunogenic and had a better prognosis and response rate to immunotherapy. These findings revealed novel events related to tumorigenesis and tumor immune microenvironment and laid the foundation for the development of splice-switching therapies for LUAD.

## Introduction

Lung cancer is the most prevailing and fatal cancer in the world, resulting in one-quarter of all cancer deaths.^1^ About 80% to 85% of lung cancers are non-small cell lung cancer (NSCLC), and the main subtypes of NSCLC are adenocarcinoma, squamous cell carcinoma, and large cell carcinoma. Before the 1990s, squamous cell carcinoma was the most common histologic subtype of lung cancer, particularly among men. Since then, the incidence of adenocarcinoma increased to be higher than that of squamous cell carcinoma. Currently, lung adenocarcinoma (LUAD) has become the dominant histologic subtype and accounted for almost half of all lung cancer deaths.^2^ The past decade has seen tremendous progress in characterizing molecular alterations in LUAD, especially the identification of druggable mutations and immune checkpoints, leading to the successful application of targeted drugs and immunotherapies in clinical settings. Despite this progress, there is still a large proportion of LUAD patients without suitable targeted therapeutic options and the 5-year relative survival rate is still around 20%.^1^ Thus, novel therapeutic strategies are urgently needed to tackle this disease.

Gaining insight into diseases and developing novel treatments require clear molecular characteristics. Currently, we have a comprehensive understanding of DNA mutations, abnormal gene expression profiles, epigenomics, and proteomics in LUAD,^3,4^ but little is known about its aberrant alternative splicing profiles. Alternative splicing is one of the most important mechanisms of posttranscriptional gene regulation in eukaryotes, which can not only lead to the generation of mRNA isoforms with distinct or opposite functions from the same gene but also convert pre-mRNAs into transcripts that are non-translated or eliminated by nonsense-mediated decay. It has been reported that alternative splicing regulates more than 90% of human genes and is a major source of protein diversity.^5^ Since alternative splicing leads to multiple functional consequences, it not only has substantial effects on physiological processes, such as development^6^ and aging,^7^ but also plays a key role in various pathological processes. Recently, it has been increasingly recognized that splicing dysregulation can lead to cancer because alternative splicing usually changes the function of translated proteins, potentially creating oncogenes or inactivating tumor suppressor genes.^8^ For instance, previous studies have reported that Cyclin D1 (CCND1) underwent alternative splicing, resulting in the generation of a special CCND1 isoform, cyclin D1b, which lacked Thr-286. Contrary to canonical cyclin D1a, cyclin D1b was highly expressed in many tumors and proved to be tumorigenic. In other words, the oncogenic cyclin D1 isoform was produced and expressed in human cancer due to alternative splicing.^9^

Except for the key role in physiological and pathological processes, alternative splicing is also an untapped source of molecular targets for RNA-based therapies that can actually be applied in clinical practice.^10^ Compared to traditional protein-based therapies, RNA-based therapies have potential advantages because they can theoretically target any gene (especially the previously undruggable targets), have more direct and simple development procedures, and are more stable at room temperature, a feature conducive to their distribution and storage.^11^ Several RNA-based therapeutic platforms have been developed, including antisense oligonucleotides (ASOs), microRNA, small interfering RNA, and aptamers.^12^ Among them, the application of ASOs to convert alternative splicing is one of the most well-established and promising disease treatment strategies.^13^ Recently, dozens of ASOs that modulate alternative splicing have been developed for the treatment of intractable diseases. For example, researchers have designed specific ASOs to reduce the expression of detrimental SMN2 isoforms while facilitating therapeutic SMN2 isoforms to inhibit spinal muscular atrophy, leading to the first splice-switching therapy (Spinraza) approved by FDA in 2016.^14^ Besides, a variety of preclinical studies and clinical trials have confirmed the great promise of ASOs in the treatment of cancer. As of January 1, 2021, a total of 229 clinical trials have studied 60 oligonucleotide drugs for the treatment of cancer, of which 195 trials have applied ASOs as interventions, including 15 phase 2/3 or phase 3 clinical trials.^15^ Nevertheless, little attention has been paid to exploring the abnormality of alternative splicing in LUAD, which would undermine our comprehensive understanding of LUAD and the development of therapeutics targeting aberrant RNA splicing.

In this study, we integrated multi-omics data to comprehensively and robustly investigate the landscape of splicing alterations in LUAD based on a total of 799 LUAD samples and 204 adjacent-normal samples. Through the perspective of RNA binding proteins, somatic mutations, and DNA methylations, we thoroughly examined the regulatory factors mediating the splicing dysregulation in LUAD. The functional relevance of the splicing dysregulation was also interrogated. In addition, we found that one LUAD subtype with particular splicing dysregulation profiles was more immunogenic and had a better prognosis and response rate to immunotherapies. This study not only provides new insights into the molecular mechanism of the occurrence and development of LUAD by revealing the characteristics of splicing dysregulation patterns, but also lays the foundation for the development of splice-switching therapies for LUAD and promotes the application of these promising therapies in clinical settings, which is expected to further improve the current poor prognosis of LUAD patients.

## Results

### Identification and statistics of aberrant alternative splicing events in LUAD

**Fig. 1:**
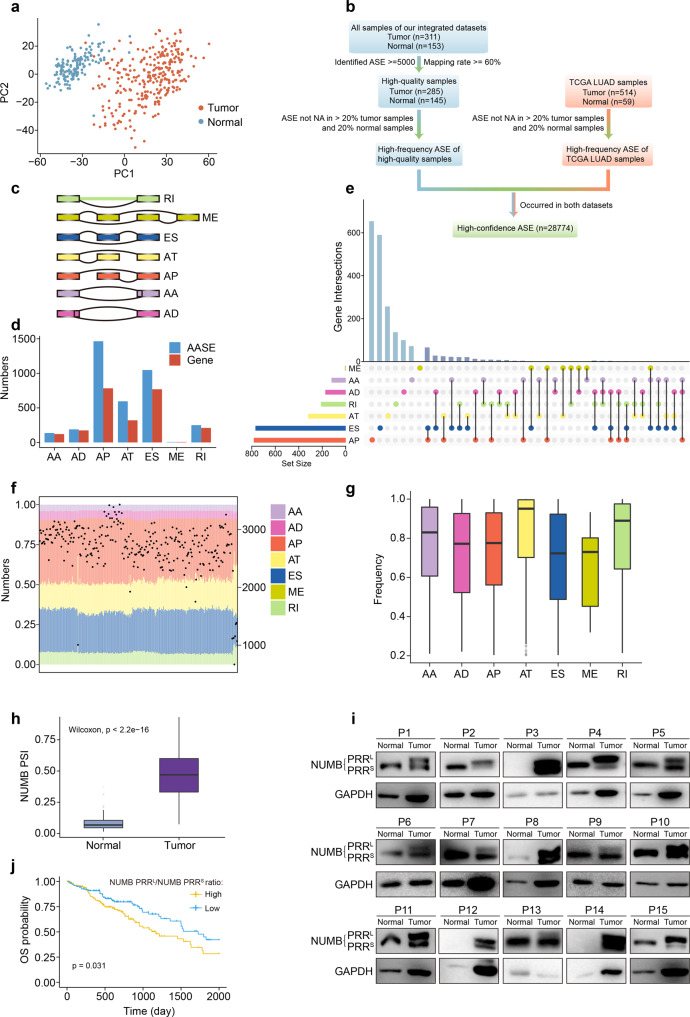
Overview of aberrant alternative splicing events (AASEs) and related genes in LUAD. **a** Principal component analysis (PCA) based on gene expression profiles of LUAD and adjacent-normal samples in our integrated datasets. Each point represents a sample. **b** The pipeline of identifying high-confidence ASEs. **c** Ideographs of the seven types of ASEs. Each box indicates an exon. Exons connected by lower lines in each ideograph indicate exclusion transcripts, while upper lines represent inclusion transcripts. **d** The number of the seven types of AASEs and related genes identified in LUAD. **e** UpSet plot demonstrating the intersections among the seven types of AASEs in LUAD. **f** Summary of AASEs in LUAD samples in our integrated dataset. Bars show the proportion of the seven ASE types in each sample while points indicate the total number of AASEs identified in each sample. **g** Box plot displaying the detection frequency of the seven types of AASEs in LUAD samples. **h** Box plot showing that the PSI values of NUMB ES event were significantly higher in LUAD samples compared to adjacent-normal samples. **i** Western blot showing the expression of NUMB PRR^L^ and NUMB PRR^S^ in LUAD samples and paired adjacent-normal samples. **j** Kaplan–Meier survival curves comparing the overall survival of high and low NUMB PRR^L^/NUMB PRR^S^ ratio subgroups. Patients were stratified into high (top 25th percentile) and low (bottom 25th percentile) subgroups based on their PSI value

To systematically reveal the AASEs in LUAD, we collected two datasets. One is our integrated cohort, including 285 tumor samples and 145 paired adjacent-normal samples with high-quality (see methods). The other is the TCGA LUAD cohort, including 514 tumors and 59 adjacent-normal samples. Principal component analysis showed that tumor and adjacent-normal samples from our integrated cohort were clearly separated into two different groups (Fig. 1a), suggesting that the quality of sequencing data was consistent and the batch effect of our integrated cohort was acceptable.

Based on our integrated cohort and the TCGA LUAD cohort, we identified 28774 high-confidence alternative splicing events (ASEs), which affected 8726 genes, including targets for tyrosine kinase inhibitors used to treat lung cancer, such as EGFR, RET, and MET (see methods) (Fig. 1b). This result supported the possibility that alternative splicing patterns might modify drug responses of LUAD patients as demonstrated by previous reports.^16,17^ In this study, seven different types of ASEs were identified, including retained intron (RI), mutually exclusive exons (ME), exon skip (ES), alternate terminator (AT), alternate promoter (AP), alternate acceptor site (AA), and alternate donor site (AD) (Fig. 1c). We further investigated aberrant alternative splicing events (AASEs) based on our integrated cohort due to the more balanced number of tumor samples and normal samples than the TCGA LUAD cohort (Supplementary Fig. 1).

We identified 3688 AASEs affecting 2081 genes, of which AP and ES events accounted for the vast majority (Fig. 1d and Supplementary Table 1). Although some genes were affected by multiple types (up to three) of AASEs concurrently, most genes were only regulated by one type of AASEs (Fig. 1e). The ratio of 7 types of AASEs was similar across LUAD patients and most patients contained more than 2000 AASEs (Fig. 1f). Among these 3688 AASEs, 1670 AASEs were also significantly aberrant in the TCGA LUAD cohort despite only 59 normal samples in this cohort as a reference, and 97% of them (1620/1670) have the same direction of upregulation or downregulation in both cohorts, suggesting the AASEs we identified were robust.

Among the 2081 genes affected by AASEs, 1981 genes belonged to protein-coding genes, indicating that AASEs intently affected coding genes and may have tremendous implications on LUAD. In addition, according to Bailey et al.’s definition of driver genes,^18^ 53 genes belonged to driver genes and were over-represented in AASEs-related genes (hypergeometric test, *P* < 0.001), further suggesting the critical role of AASEs in LUAD. Furthermore, most AASEs can be identified in at least 75% of LUAD samples (Fig. 1g), implying that therapeutics targeting AASEs may benefit a lot of LUAD patients.

One of the most significant AASEs in LUAD was the exon skipping event of NUMB (Supplementary Fig. 2a). This event generates two major NUMB isoforms: the long isoform NUMB PRR^L^ and the short isoform NUMB PRR^S^. They differ in the length of their proline-rich region (PRR), due to the inclusion or exclusion of a 48 amino-acids insert encoded by the skipping exon (Supplementary Fig. 2b). It has been reported that NUMB PRR^L^ promotes cell proliferation while NUMB PRR^S^ directs cell differentiation.^19^ Besides, NUMB PRR^L^ increases the proliferation and metastasis of liver and breast cancer, while NUMB PRR^S^ inhibits these phenotypes.^20,21^ The inclusion of skipping exon of NUMB (NUMB PRR^L^) was significantly increased in LUAD compared to adjacent-normal tissues (Fig. 1h). The western blotting experiment further validated that the NUMB PRR^L^ / NUMB PRR^S^ ratio was significantly increased in LUAD samples (Fig. 1i). Furthermore, survival analyses showed that a high NUMB PRR^L^ / NUMB PRR^S^ ratio indicated poor overall survival (OS) (Fig. 1j) and progression-free survival (PFS) (Supplementary Fig. 2c), while the NUMB expression level did not associate with OS and PFS (Supplementary Fig. 2d, e). These findings suggested that the aberrant alternative splicing of NUMB may contribute to the malignancy of LUAD. Further studies on the molecular mechanism and intervention value of this AASE in LUAD are required in the future.

### The regulatory pattern of RNA binding proteins (RBPs) on AASEs in LUAD

**Fig. 2:**
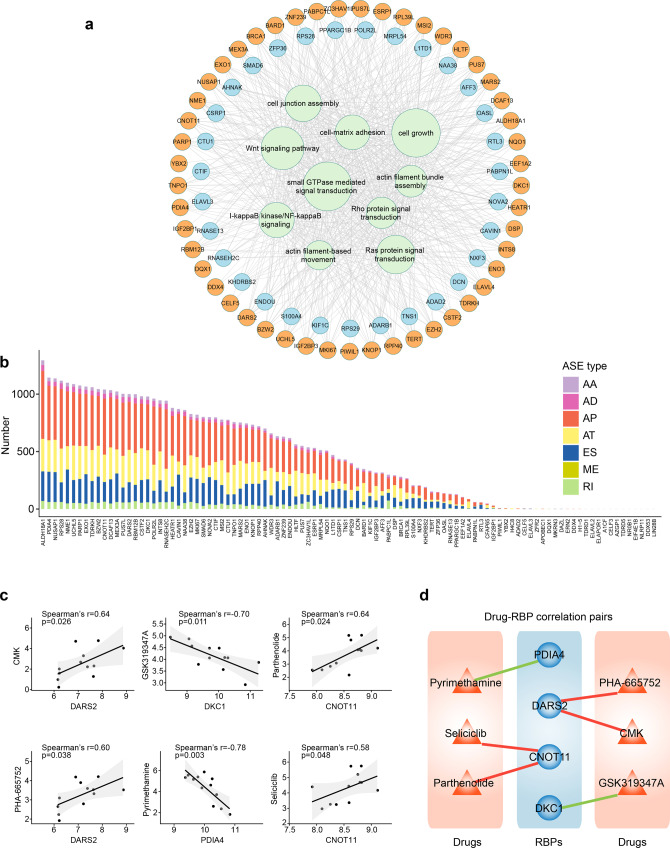
Regulation of AASEs by RNA binding proteins (RBPs). **a** The splicing-regulatory network illustrates the biological processes (green circles) enriched by AASEs-affected genes regulated by differentially expressed RBPs. The size of green circles stands for the number of regulated genes in each Gene Ontology term. The orange and blue circles represent high and low expressed RBPs in LUAD, respectively. **b** The number of AASEs regulated by each differently expressed RBP in LUAD. **c** Dot plots showing the correlations between the expression levels of RBPs and IC50 values of corresponding drugs. **d** Regulatory networks summarizing the Drug-RBP correlation pairs, blue circles indicating drug-related RBPs, orange triangles representing drugs tested in LUAD cancer cell lines, red lines and green lines standing for positive and negative correlations, respectively

One of the most common regulators of ASEs is RBPs, the disturbance of which would lead to extensive AASEs. These regulators are a rich resource of candidate targets for cancer treatments, as demonstrated by one recent work that aimed to target RBPs in acute myeloid leukemia.^22^ To comprehensively unveil the perturbation of RBPs and their role on AASEs in LUAD, we identified differentially expressed RBPs and assessed the relationship between these differentially expressed RBPs and AASEs. As a result, one hundred differentially expressed RBPs were detected, in which 68 RBPs were upregulated and 32 RBPs were downregulated in LUAD (Supplementary Table 2), which was consistent with previous studies suggesting that RBPs tended to be upregulated in cancer.^23^ In addition, we noticed that 88 differentially expressed RBPs were significantly associated with 2505 AASEs, resulting in 46704 RBP-ASE pairs. About half (47%) (21928/46704) pairs were negatively correlated (the higher the RBP level, the lower the inclusion level of the corresponding alternative exon), and another half were positively correlated, suggesting the regulation of AASEs by RBPs was balanced on the whole (Supplementary Table 3). We established a dysregulation network based on the significant correlation between RBPs and AASEs. This network illustrated that AASEs-related genes affected by differentially expressed RBPs were enriched in processes and pathways crucial for tumorigenesis, such as “Ras protein signal transduction”, “NF-kappaB signaling pathway”, “Wnt signaling pathway”, and for invasion and metastasis, such as “cell-matrix adhesion” and “actin filament-based movement” (Fig. 2a and Supplementary Table 4), suggesting these differentially expressed RBPs played a critical role in tumor development through causing splicing alterations in LUAD. Interestingly, it was largely disparate for the number of AASEs regulated by each RBP, and ALDH18A1, PDIA4, and NUSAP1 were the RBPs regulating the greatest number of AASEs (Fig. 2b).

To measure the effect of AASE-regulated RBPs on drugs in LUAD, we examined the correlation between the expression of the top 20 RBPs with the highest regulatory capabilities and drug activities across LUAD cell lines. As a result, we found that four upregulated RBPs in LUAD were associated with drug sensitivities. In detail, the DARS2 gene was positively correlated with the IC50 value of CMK and PHA-665752 drugs, and the CNOT11 gene was positively related to the IC50 value of Parthenolide and Seliciclib drugs, suggesting the effects of these drugs may be inhibited by the high expression of DARS2 and CNOT11 in LUAD. Interestingly, the DKC1 gene was negatively correlated with the IC50 value of GSK319347A, a dual inhibitor of TBK1 and IKKε, and the PDIA4 gene was negatively associated with the IC50 value of Pyrimethamine, a STAT3 inhibitor displaying anti-cancer and immune-stimulatory effects,^24^ suggesting that the high expression of DKC1 and PDIA4 in LUAD may increase the sensitivity of GSK319347A and Pyrimethamine, respectively (Fig. 2c, d).

**Fig. 3:**
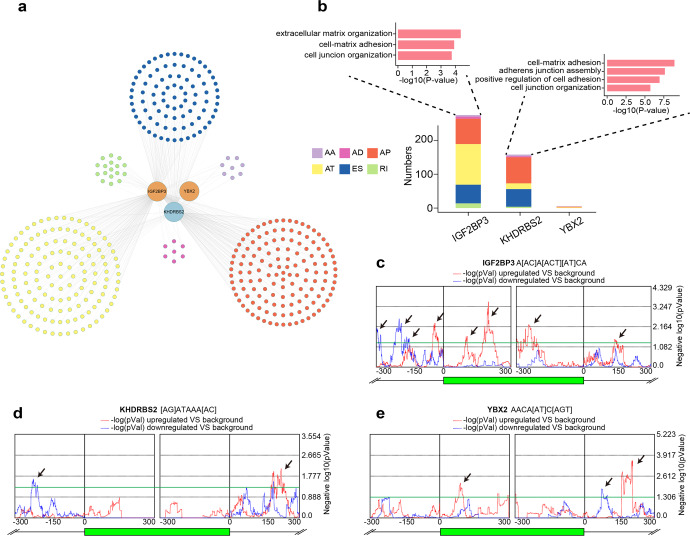
More robust regulatory effects of RBPs on AASEs. **a** The more reliable splicing-regulatory network. The orange and blue circles indicate high and low expressed RBPs in LUAD, and the colored dots represent AASEs grouped by seven types. **b** The number of AASEs affected by three RBPs. Pink bars indicate the GO terms significantly enriched by those regulated AASEs. **c**–**e** Motif search analysis for IGF2BP3 (**c**), KHDRBS2 (**d**), and YBX2 (**e**) binding sites around alternative spliced exons. Arrows indicate peaks of significant over-enrichment. The colored dashed lines represent the degree of over-enrichment of motifs within upregulated (red) and downregulated (blue) exons as demonstrated by negative log10 (*p* value). The green horizontal line is set at *p* = 0.05

In order to modulate ASEs directly, most RBPs have to bind their corresponding motifs near splicing sites. We further applied DeepBind to investigate whether the ±300 bp regions around splicing sites of AASEs can be bound by three differentially expressed RBPs (IGF2BP3, KHDRBS2, and YBX2). Based on the results from DeepBind and the significant RBP-ASE pairs, we built a more robust but smaller dysregulation network (Supplementary Table 5). This network reflected that three RBPs mediated 438 AASEs in LUAD, of which AT, AP, and ES accounted for the most (Fig. 3a). Among these RBPs, IGF2BP3 mediated the greatest number of AASEs, and genes affected by these AASEs were enriched in tumor invasion and metastasis processes, such as “extracellular matrix organization”, “cell junction organization”, and “cell−matrix adhesion”. AASEs regulated by KHDRBS2 were also enriched in similar biological processes (Fig. 3b). To identify the position of motifs and validate that the motifs of these three RBPs were enriched in regions around splicing sites of AASEs, we identified a control set of non-differentially cassette exons (*n* = 9300), a set of upregulated cassette exons (*n* = 543), and a set of downregulated cassette exons (*n* = 506). We then performed motif scanning analyses for the transcript sequences flanking the differential cassette exons, comparing against the non-differentially cassette exons. These analyses indicated that the well-conserved binding motif for IGF2BP3 was significantly over-represented within the upregulated cassette exonic regions and intronic regions within 150 bp near upstream and downstream splicing sites. Well-conserved binding motif for IGF2BP3 was also significantly over-represented in intronic regions about 150–300 bp upstream of downregulated cassette exons (Fig. 3c). For KHDRBS2, the binding motif was significantly enriched in intronic regions about 150–300 bp upstream of downregulated cassette exons, and in intron regions about 150–300 bp downstream of upregulated cassette exons (Fig. 3d). We also observed a significant over-enrichment for YBX2 binding motifs within the upregulated exons and around 150 bp downstream of upregulated exons. Well-conserved binding motif for YBX2 was also significantly over-represented in intron regions about 0–150 bp downstream of downregulated cassette exons (Fig. 3e). Thus, motif searching analysis further confirmed the regulatory role of these three RBPs on AASEs. Taken together, these results comprehensively revealed the regulatory role of RBPs on AASEs in LUAD, suggested that RBPs disturbed alternative splicing profiles with key functional meaning in LUAD, and provided important information for the development of novel therapeutic strategies.

### The regulatory pattern of DNA mutation and methylation on AASEs in LUAD

**Fig. 4:**
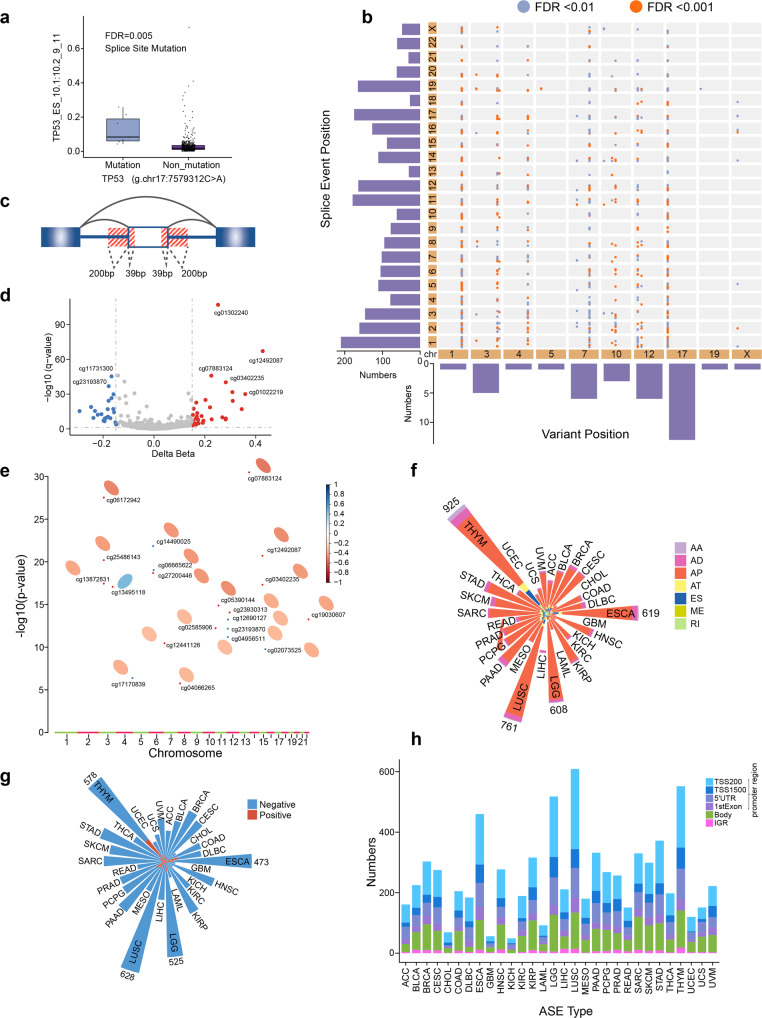
Regulatory pattern of DNA mutation and methylation on AASE in LUAD. **a** Box plot showing the ES event of TP53 were regulated by the splice site mutation of TP53. **b** Overview of the trans-sQTLs. The x-axis indicates the mutation positions and the y-axis indicates the splice sites across chromosomes. Bars vertical to the x-axis summarize the number of trans-sQTLs in each chromosome and bars parallel to the x-axis summarize the regulated ASEs within each chromosome. **c** Schematic plot showing the boundary regions of alternative exons. **d** Volcano plot showing the differential methylated CpG sites within the boundary regions of alternative exons in LUAD. **e** Summary of the 21 differentially methylated CpG sites that regulated their corresponding AS events. The X-axis represents the position of CpG sites across chromosomes and the Y-axis indicates the significance of correlation coefficients between CpG sites and corresponding ASEs. Red and blue dots stand for highly and lowly methylated CpG sites in LUAD, respectively. Ellipse indicates a positive (orange) or negative (blue) relationship between methylated levels of the CpG sites and the inclusion rate of corresponded alternative exons. **f** The number of seven types of AASEs regulated by methylated CpG sites across 30 cancer types. **g** The number of AP events positively (red) and negatively (blue) regulated by methylated CpG sites across 30 cancer types. **h** Bar plot showing the number of methylated CpG sites that regulated AP events in promoter regions, gene body regions, and intergenic regions (IGR) across 30 cancer types

It has been reported that DNA mutations might also influence ASEs through altering the regulatory RNA sequences, the structure of RBPs, or other regulatory molecules. Single nucleotide variants (SNV) having regulatory impacts on ASEs are called splicing quantitative trait loci (sQTL). Here, the “MatrixEQTL” algorithm was applied to uncover sQTLs in LUAD. Generally speaking, if a regulatory SNV is within 100 Kbp around its’ corresponding splicing site, this SNV is defined as cis-sQTL; otherwise, it is trans-sQTL. As a result, only one cis-sQTL was identified where a mutation in the splice site of TP53 would increase the inclusion level of exon 10 (Fig. 4a). We also found 36 trans-sQTLs regulating widespread ASEs in LUAD. Interestingly, most trans-sQTLs in LUAD belonged to missense mutations (28/36) (hypergeometric test, *P* < 0.001), further justifying the regulatory effects of these SNVs on alternative splicing. In LUAD, trans-sQTLs were located on chromosomes irregularly. Thirteen chromosomes had no trans-sQTLs located, while 13 trans-sQTLs on chr17 and all were located in the TP53 gene. The distribution of ASEs regulated by trans-sQTLs was also uneven. More than 200 ASEs on chr1 but less than 50 ASEs on chr13, chr18, and chr21 were regulated by trans-sQTLs (Fig. 4b). These results comprehensively unraveled the pattern of sQTLs and helped us better understand the complex genetic architecture of LUAD.

In addition to DNA mutations, epigenomic studies have revealed that DNA methylation may also play a role in splicing regulation via methyl-CpG binding protein 2 and heterochromatin protein 1.^25,26^ To examine whether AASEs were partially caused by disturbed DNA methylation levels, we first identified 1980 CpG sites at boundaries of alternatively spliced exons (Fig. 4c). The region of exon boundaries was defined based on the finding that splicing cis-regulatory elements are most likely in the size of 200 nucleotides for intronic regions and 39 nucleotides for exonic regions around the splice sites of exons.^27^ After comparing these CpG sites between LUAD and adjacent-normal tissues, we identified 56 CpG sites with differential methylation levels (Fig. 4d). We then investigated the regulatory role of these differentially methylated CpG sites on their corresponding AASEs and found that 21 CpG sites were significantly correlated with AASEs. Interestingly, except for one, all of these CpG sites were negatively correlated with their corresponding AASEs, and all these AASEs belonged to AP (alternative promoters, also known as alternative first exons) (Fig. 4e). In another word, the higher the methylation levels of these CpG sites around the boundary of the alternative first exon, the lower the usage of this alternative promoter. To investigate whether this rule was prevalent in cancer, we analyzed the relationship between CpG sites and their corresponding ASEs in 30 other types of cancer. As a result, the vast majority of ASEs regulated by CpG sites were AP events (Fig. 4f) and the inclusion level of most AP events was negatively correlated with the methylation level of corresponding CpG sites (Fig. 4g). Besides, most CpG sites that have a regulatory effect on AP events were located in the promoter region and a small part were in gene body regions (Fig. 4h). All these results suggested that the primary role of DNA methylation on ASEs in cancer is to inhibit the usage of alternative promoters.

### The functional implications of AASEs in LUAD

**Fig. 5:**
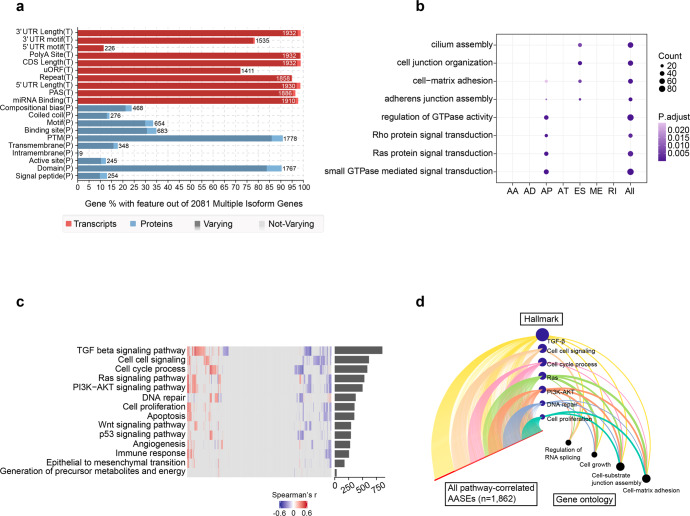
Functional implications of AASEs. **a** The number and percentage of genes with each feature (indicated on the y-axis) among the 2081 AASEs-related genes. The dark color in each bar represents the percentage of genes having isoforms that each feature varies. **b** GO enrichment analysis result for AASEs-related genes. **c** Heatmap of the correlation coefficients of the AASEs correlated with 14 cancer hallmarks. Each column represents a single AASE and each row depicts the results of the correlation to a single hallmark signature. The color represents the strength and direction of the correlation (red indicating positive; blue indicating negative) of a single AASE with each hallmark. Columns are sorted by hierarchical clustering. Rows are ranked by the total number of AASEs correlates passing statistical criteria for each hallmark. The number of AASEs significantly correlated with each hallmark was represented in the bar chart. **d** Hive plot depiction of AASEs correlated with 14 cancer hallmarks and the biological processes associated with genes affected by AASEs. All hallmarks-related AASEs are displayed on the left axis. The top seven hallmarks with the most correlated AASEs are represented as nodes on the middle axis. The size of these nodes reflects the number of AASEs correlated with each hallmark. The right axis indicates four summary gene ontology terms. The width of the edges connecting the nodes on the middle axis to the nodes on the right axis is proportional to the −log10 (*p* value) of the enrichment result. The size of the nodes on the right axis is proportional to the total number of hallmarks associated with each biological process

So far, we have comprehensively uncovered the upstream regulators of AASEs in LUAD, but the functional implications of these AASEs are still unexplored. Revealing the biological relevance of AASEs in LUAD can point us to promising directions when designing splice-switching therapeutics.^28^ Here, we found that for 2081 genes affected by AASEs, most have isoforms with varying features at the transcript levels, such as CDS length, 3′UTR length, as well as at the protein levels, such as protein domains. These results suggested that aberrant splicing of these genes may lead to considerable functional changes (Fig. 5a). To further unravel the biological effects of AASEs on LUAD, we conducted gene ontology (GO) enrichment analysis on genes affected by AASEs. As a result, we noticed that among seven types of ASEs, only genes affected by aberrant AP and ES events were significantly enriched in cancer-associated GO terms. Interestingly, aberrant AP events and ES events exerted impacts on distinct biological processes. To illustrate, aberrant AP events primarily affected “small GTPase mediated signal transduction”, such as the “Ras signaling pathway” and “Rho signaling pathway”, which were well-known pathways boosting tumor proliferation. Aberrant ES events mainly affected tumor metastasis-related biological processes, such as “cilium assembly”,^29^ “cell junction organization” and “cell−matrix adhesion” (Fig. 5b). To investigate the functional implications of AASEs more deeply, we measured the activation degree of 14 tumor hallmarks through the Single-sample Gene Set Enrichment Analysis (ssGSEA) method and examined the relationship between AASEs and the activation degree of these hallmarks. After filtering correlation pairs based on correlation coefficient (|r| > 0.3) and false-discovery rate (FDR < 0.05), each hallmark was significantly associated with 34–857 AASEs (Supplementary Table 6). The hallmarks mostly affected by AASEs were “TGF beta signaling pathway”, “cell signaling”, “cell cycle process”, and “Ras signaling pathway”. However, “EMT processes” and “generation of precursor metabolites and energy” were scarcely affected (Fig. 5c). We further investigated the biological processes enriched by the AASEs associated with cancer hallmarks and employed the hive plot to exhibit how AASEs (left axis) associated with cancer hallmarks (middle axis) and the functional enrichment of genes affected by these AASEs (right axis). We found that AASEs associated with many hallmarks were also significantly enriched in “cell-substrate junction assembly” and “cell-matrix adhesion” (Fig. 5d). These results further suggested that AASEs in LUAD had substantial functional implications on biological processes relating to tumor proliferation and metastasis.

### LUAD subtype classification based on AASEs showed distinct biological characteristics

**Fig. 6:**
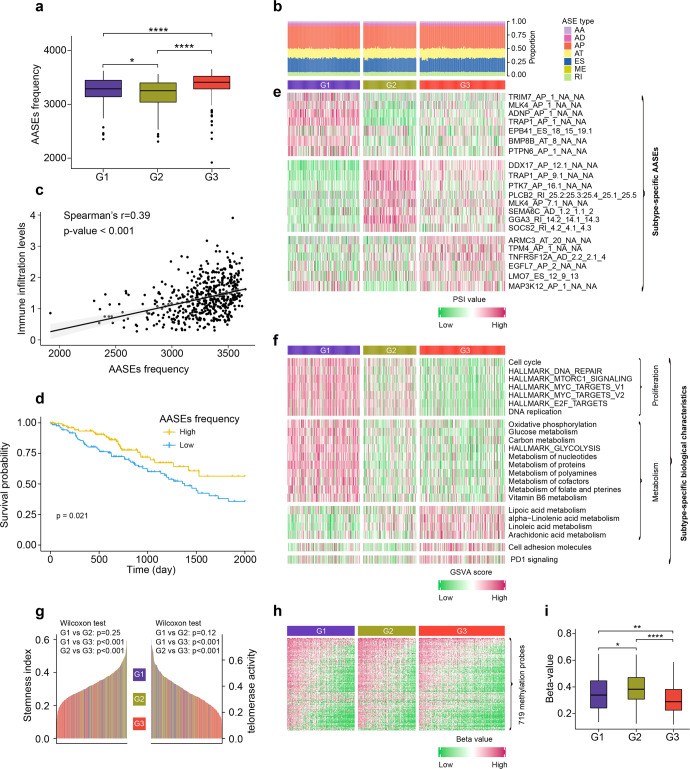
The AASEs and biological characteristics of the three LUAD subtypes. **a** Box plot showing the AASEs frequency of LUAD samples in each subtype. **b** Bar plot showing the proportion of the seven AASEs types in each LUAD sample. **c** AASEs frequency was positively correlated with immune cell infiltration levels. **d** Kaplan–Meier overall survival curves comparing AASEs frequency high and low subgroups in LUAD. Patients were stratified into high (top 25th percentile) and low (bottom 25th percentile) subgroups based on their AASEs frequency. **e** Heatmap representing the subtype-specific AASEs in each LUAD subtype. **f** The subtype-specific biological characteristics of each AASE subtype. **g** Bar plots showing the stemness index and telomerase activity of each LUAD sample. **h**, **i** Heatmap and box plot showing the methylation levels of 719 most variable DNA methylation-specific probes in CpG island promoter regions

Molecular subtyping based on RNA splicing patterns has been shown to stratify patients with different prognosis or treatment efficacy in other tumor types,^30–32^, and thus could be leveraged to improve patient stratification in LUAD. Based on 3688 AASEs, we split 514 TCGA LUAD samples into three subtypes through consensus clustering analysis: G1 (*n* = 176, 34.2%), G2 (*n* = 130, 25.3%), and G3 (*n* = 208, 40.5%) (Supplementary Fig. 3a–c and Supplementary Table 7). We noticed that the total number of AASEs (AASEs frequency) was the highest in G3 compared to G1 and G2 (Fig. 6a). The proportion of seven types of AASEs was similar among the three subtypes (Fig. 6b), which was concordant with Fig. 1f. Potential links between AASE frequency and gene alterations were found. For example, the non-silent mutations of splicing factors CWC22, ELAVL1, and PRPF40B were associated with high AASEs frequency (Supplementary Fig. 4a–c), while the non-silent mutations of splicing factors RNF20 and MBNL2 were related to low AASEs frequency (Supplementary Fig. 4d, e). Almost all samples with non-silent mutation of U2AF1 or SF3B1 or RBM10 or SRSF2 have AASEs larger than 3000 (Supplementary Fig. 4f). What’s more, high AASEs frequency indicated high levels of immune cell infiltration as well as a good prognosis in LUAD (Fig. 6c, d).

The three AASE subtypes of LUAD had specific AASEs characteristics and patterns. For example, the AP events of several tumor-related genes, such as TRIM7, MLK4, ADNP, and TRAP1, had higher inclusion levels in G1. AASEs of tumor-related genes, such as DDX17, PLCB2, and SEMA6C had higher inclusion levels in G2. While AASEs of tumor-related genes, such as ARMC3, TPM4, and TNFRSF12A had higher inclusion levels in G3 (Fig. 6e and Supplementary Table 8).

We further investigated the biological characteristics of the three subtypes and found that G1 was a quick-proliferation subtype, with pathways enriched in “Cell Cycle”, “DNA Repair”, “DNA replication”, and so on (Fig. 6f). G1 was also a high-metabolism subtype, with pathways enriched in “Oxidative Phosphorylation”, “Glucose metabolism”, “Metabolism of proteins”, “Metabolism of nucleotides”, and so on (Fig. 6f). These results suggested that G1 may get more benefit from the conventional chemotherapies targeting highly proliferative cells. G3 had a specific metabolism pattern, characterized by a preference for fatty acid metabolism (Fig. 6f). Besides, G3 was a subtype having high cell adhesion molecules, PD1 signaling (Fig. 6f) as well as low stemness index and telomerase activity (Fig. 6g). In addition, from the landmark LUAD paper,^33^ we collected 719 most variable DNA methylation-specific probes in CpG island promoter regions that were used to identify LUAD methylation subtypes. According to these probes, G2 was methylation-high while G3 was methylation-low (Fig. 6h, i).

### AASE subtypes of LUAD showed distinct immune characteristics

**Fig. 7:**
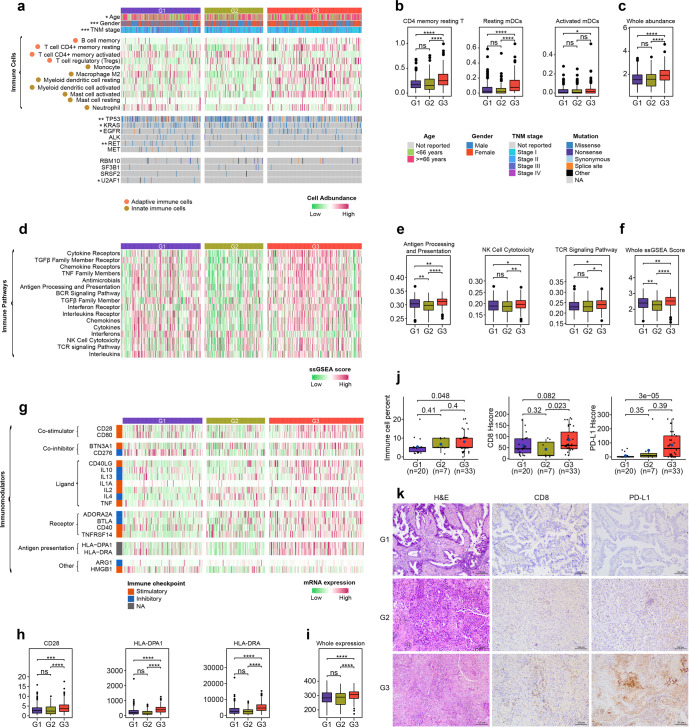
The immune characteristics of AASE subtypes of LUAD. **a** Heatmap showing the infiltration levels of immune cells that are significantly different in the three subtypes. Fisher’s exact test was used for categorical variables: age, gender, TNM stage, and status of TP53, KRAS, EGFR, ALK, RET, and MET mutations and splicing factors RBM10, SF3B1, SRSF2, and U2AF1 mutations. **b**, **c** Box plot showing the infiltration levels of CD4 memory resting T cells, resting mDCs, activated mDCs (**b**), and total immune cells (**c**) in the three subtypes. *P < 0.05, **P < 0.01, ***P < 0.001, ****P < 0.0001, ns indicating no significance. **d** The activation degree of immune pathways showed a significant difference among the three subtypes. **e**, **f** Distribution of the intensity of “Antigen Processing and Presentation”, “NK Cell Cytotoxicity”, “TCR signaling pathway” (**e**), and the whole intensity of all immune pathways (**f**) in the three subtypes. **g** Heatmap showing the expression of significantly different immunomodulators in the three subtypes. **h**, **i** Distribution of the expression of CD28, HLA-DPA1, and HLA-DRA (**h**), and total immunomodulators (**i**) in the three subtypes. **j** Box plot showing the levels of immune cell infiltration, CD8 expression, and PD-L1 expression in the three subtypes, which were evaluated through the H&E-stained and IHC-stained slides of 60 LUAD samples in our own cohort. The blue dot in each box represents the mean value. **k** H&E and IHC (CD8 and PD-L1) staining of representative LUAD samples belonging to G1, G2, and G3, respectively. Scale bars, 100 μm

Since the AASEs frequency was positively correlated with immune cell infiltration levels, we further deeply characterized the immune characteristics of AASE subtypes. Firstly, we evaluated and compared the infiltration levels of 22 immune cell types among the three subtypes (Supplementary Table 9). As a result, 11 cell types showed significant differences including seven types of innate immune cells and four types of adaptive immune cells (Fig. 7a). Most cell types exhibited the highest infiltration levels in the G3 subtype, such as CD4 + memory resting T cells and myeloid dendritic cells (Fig. 7b). The infiltration level of all immune cells was also the highest in G3 (Fig. 7c).

Another criterion to measure the immune microenvironment is the activation state of immune-related pathways. We quantified the activation degree of 17 immune pathways in each tumor sample through ssGSEA analysis (Supplementary Table 10) and found that 16 pathways exhibited significant differences among the three subtypes (Fig. 7d). Many immune pathways critical for suppressing tumor growth were consistently upregulated in G3, such as “Antigen processing and presentation”, “NK cell cytotoxicity”, and “TCR signaling pathway” (Fig. 7e). Consistent with the infiltration level of immune cells, the activation level of all immune pathways within the tumor microenvironment was the highest in G3 (Fig. 7f).

To make the evaluation of the immune microenvironment more comprehensive, we further examined 78 immunomodulators in each tumor and noticed that the expression levels of 19 immunomodulators were significantly different among the three subtypes (Fig. 7g). Most differentially expressed immunomodulators were the highest in the G3 subtype, including important co-stimulators (CD28 and CD80) and genes for antigen presentation (HLA-DPA1 and HLA-DRA) (Fig. 7h). The total expression of 78 immunomodulators was also the highest in G3 (Fig. 7i).

To validate the immune cell signatures in each tumor sample, we further employed TIMER2.0 to estimate the immune infiltration levels. The immune microenvironment measured by EPIC, MCP-counter, quanTIseq, TIMER, and xCell algorithms all suggested that the immune cell signatures were the highest in G3 (Supplementary Fig. 5a–f). These results consistently suggested that AASEs patterns were related to the immune microenvironment of LUAD and the G3 subtype was more immunogenic among all LUAD samples.

To investigate whether the AASE subtypes of LUAD were general, we did a similar analysis on our own cohort. Ninety-six LUAD samples in our own cohort were split into three groups with different AASE profiles: G1 (*n* = 42, 43.75%), G2 (*n* = 7, 7.29%), and G3 (*n* = 47, 48.96%) (Supplementary Table 11). As a result, the AASEs characteristics and the biological characteristics of each subtype in our own cohort were similar to the corresponding subtype identified in the TCGA LUAD cohort (Supplementary Fig. 6a–c). Besides, similar to the TCGA LUAD cohort, the levels of immune cell infiltration, immune pathways activation and immunomodulators expression were consistently the highest in the G3 subtype, suggesting that our identification of the AASE subtypes of LUAD was robust and reliable (Supplementary Fig. 6d–i). To experimentally validate the immune characteristics of each subtype, two experienced pathologists independently evaluated the proportion of infiltrated immune cells on H&E-stained slides of 60 LUAD samples in our own cohort. As expected, the proportion of infiltrated immune cells was the highest in G3, suggesting that G3 was indeed “hot” tumors (Fig. 7j, k and Supplementary Table 12). We also performed immunohistochemical (IHC) staining of CD8 and PD-L1 and evaluated their expression levels using Histoscore (H-score). H-score takes the proportion of positive cells (0–100%) and the average intensity of the positive staining (0, 1+, 2+, or 3+) into consideration. Representative images corresponding to each intensity level were shown in Supplementary. Fig. 6j. We found that CD8 and PD-L1, the key immune markers and predictors of immunotherapy efficacy, were the highest in G3 (Fig. 7j, k and Supplementary Table 12).

### The clinical implications of AASE subtypes of LUAD

**Fig. 8:**
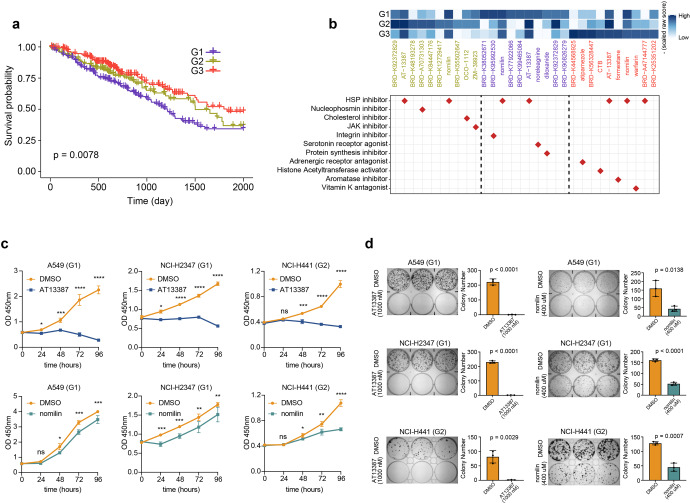
The clinical implications of AASE subtypes of LUAD. **a** Kaplan–Meier curve showing the survival probability of LUAD patients grouped by the three LUAD subtypes. **b** Upper panel showing the top ten compounds that reverse the expression profiles of each LUAD subtype. Lower panel shows the action mechanism of each drug. **c** Growth curves of LUAD cell lines treated with AT-13387 and nomilin. Representative data from six biological repeats were shown (mean ± SD). **d** Colony formation assays of three LUAD cell lines treated with DMSO or both drugs as indicated

In general, the immunogenic microenvironment implies a good prognosis and a good response rate to immune checkpoint blockades. Due to the consistently higher immune infiltration of the G3 subtype, we wondered whether this subtype had better survival than the other two subtypes. As expected, G3 had the best prognosis and could serve as an independent prognostic factor (Fig. 8a and Supplementary Fig. 7a). To further investigate whether G3 had a better response rate to immune checkpoint blockades, we applied the TIDE algorithm to predict the response of each LUAD sample to immunotherapy and observed that G3 had the highest response rate (Chi-square test, *p* = 6.966e-07) (Supplementary Fig. 7b). Taken together, based on the AASEs profiles, we found one subtype of LUAD with an immunogenic microenvironment that has a better prognosis and response rate to immunotherapy.

One of the most important applications of molecular subtype is to stratify patients for precision medicine. To measure whether the three LUAD subtypes were sensitive to different drug profiles, we employed Connectivity Map (Cmap), a data-driven method for uncovering relationships among diseases, chemical compounds, and genes, to look for potential drugs that might be useful against each LUAD subgroup. We found that different LUAD subtypes were sensitive to distinct compounds, further confirming the significance of molecular subtyping based on distinct AASEs profiles. Noticeably, although the three LUAD subtypes were sensitive to distinct drugs that target different pathways, they were all sensitive to HSP inhibitors, such as AT-13387 and nomilin (Fig. 8b). To experimentally validate this finding, we first downloaded the raw RNA sequencing data of 55 LUAD cell lines derived from the Cancer Cell Line Encyclopedia (CCLE). For each cell line, we combined this cell line with the TCGA LUAD cohort to perform the consensus clustering analysis to determine which subtype each cell line belonged to. As a result, among 55 LUAD cell lines, 49 belonged to G1, 6 belonged to G2, while no cell lines belonged to G3 (Supplementary Table 13). Then, we selected LUAD cell lines A549 (G1), NCI-H2347 (G1), and NCI-H441 (G2) to validate the efficacy of AT-13387 and nomilin. The inhibitory effect of both drugs on HSP90 in cell lines were determined by a western blot experiment, which showed that the protein expression levels of HSP90AB1 were reduced after adding AT-13387 and nomilin (Supplementary Fig. 7c). Through the cell proliferation experiments, we confirmed that both drugs could significantly inhibit the proliferation of LUAD cell lines A549, NCI-H2347, and NCI-H441 (Fig. 8c), and the inhibitory ability was positively related to the drug concentration (Supplementary Fig. 7d). The effects of these two drugs on cell growth were further confirmed using a colony formation assay (Fig. 8d).

We further interrogated the association between AASE subtypes and known subgroups defined by other researchers. We observed that our novel AASE subtypes were correlated to the published transcriptome subtypes of LUAD defined by The Cancer Genome Atlas Research Network.^33^ In detail, most samples in the G3 subtype were the terminal respiratory unit (TRU) transcriptome subtype (69/100, 69%). G1 mainly belonged to the proximal proliferative (PP) subtype and proximal inflammatory (PI) subtype (86/98, 87.8%), while G2 was dispersed in TRU, PP, and PI (chi-square test, *p* < 0.001) (Supplementary Fig. 8a). As reported by The Cancer Genome Atlas Research Network, the TRU subtype membership was prognostically favorable while PP and PI subtypes had worse prognoses.^33^ This justified the best prognosis of G3 and the worst prognosis of G1 in our study. Furthermore, we compared the AASE subtypes with the immune subtypes defined by ref. ^34^ We observed that most samples in the G3 subtype were C3 (Inflammatory) immune subtype that had the best prognosis among all six immune subtypes and high levels of immune cell infiltration (111/196, 56.6%) (chi-square test, *p* < 0.001) (Supplementary Fig. 8b), further justifying the best prognosis and the highest immune cell infiltrations of G3 among the three AASE subtypes. In addition, we analyzed the correlation between AASE subtypes and the tumor microenvironment (TME) subtypes defined by ref. ^35^ We found that G3 had the highest proportion of immune-enriched (IE) and immune-enriched, fibrotic (IE/F) subtypes compared to G1 and G2 (53.4% for G3; 45% for G2; 27.9% for G1) (chi-square test, *p* < 0.001) (Supplementary Fig. 8c). The IE and IE/F subtypes showed better OS in non-small cell lung cancer patients treated with anti-PD-L1 therapy, further suggesting that G3 may get more benefit from immunotherapies. These results consistently indicated that AASE subtypes can not only reflect the transcriptomic characteristics of the tumor parenchyma but also reflect the tumor immune microenvironment.

## Discussion

As demonstrated by Lee et al.^28^, a clear insight into splicing dysregulation in cancer would promote our understanding of tumor pathogenesis and nominate several novel therapeutic strategies. Previous research investigating alternative splicing in LUAD only used transcriptomics to explore survival-associated ASEs,^36–38^ which ignored the aberrant splicing programs that are critical for tumorigenesis and the development of new therapeutics. To the best of our knowledge, this study is the first to apply multi-omics to reveal the landscape of AASEs in LUAD. Through integrating LUAD and adjacent-normal samples from a wide range of populations, including Caucasian, Korean, Chinese, and African Americans, we obtained a comprehensive and robust repertoire of AASEs in LUAD. These AASEs primarily affected coding genes and multiple driver genes, which was consistent with a previous study on esophageal cancer,^39^ indicating that the disturbance of ASEs may have a huge impact on LUAD.

Revealing the regulators of AASEs would make us understand why extensive AASEs occurred in LUAD. Besides, The regulators of AASEs are rich sources of splice-switching therapeutic targets.^28^ Here, we systematically investigated the regulators of AASEs in LUAD, from the perspective of the splicing-regulatory proteins, somatic mutations, and DNA methylations. About 100 differentially expressed RBPs were identified and 88 RBPs exerted impacts on AASEs that were crucial for tumorigenesis and metastasis. Previous studies have reported that RBPs can be tumor driver genes by disturbing a wild range of ASEs. For example, Warzecha et al.^40^ found that ESRP1 regulated the epithelial-mesenchymal transition process by promoting epithelial splicing programs in breast cancer and suggested this RBP was a potential candidate for splicing-targeted therapies. In this study, we revealed that more than 200 AASEs were affected by ESRP1, which expressed significantly higher in LUAD than adjacent-normal samples, suggesting a critical role of this RBP in LUAD. Previous studies reported that IGF2BP3 is critical for tumorigenesis and associated with poor patient survival.^41,42^ Here, we noticed that IGF2BP3 affected cell-matrix adhesion through disturbing alternative splicing profiles in LUAD. Thus, IGF2BP3 is an attractive therapeutic target as demonstrated in this study and other researches.^43^

In addition, we found that DNA mutations regulated extensive AASEs primarily through trans-regulatory mode and most trans-sQTLs belonged to missense mutations. Especially, we noticed that the hotspot mutation of TP53 would affect a wild range of alternative splicing of other genes in LUAD, which is difficult to understand at first glance. However, one recent study reported that mutant TP53 increases the expression of splicing regulator hnRNPK,^44^ justifying the trans-sQTLs role of TP53 mutations in LUAD.

The usage of alternative exons can be enhanced or suppressed by DNA methylation in a context-specific manner. For example, one research reported that exon methylation promotes its selective usage in human normal tissues,^45^ while the other study suggested that in mouse embryonic stem cells, some DNA methylation would inhibit the usage of exons.^46^ Here through pan-cancer analysis, we found that the primary role of DNA methylation in regulating ASEs in cancer is that methylation sites in gene promoters and body regions around first exons would inhibit the usage of the alternative promoters. Many genes have alternative promoters, typically located upstream of the translation start site but also commonly existed within gene bodies.^47^ Through investigating human and mouse normal brain tissues, one study also suggested that intragenic DNA methylation abolished the activity of alternative promoters of SHANK3 in a tissue- and cell-type-specific manner.^48^ These results we found further deepen our understanding of the whole landscape of regulatory effects of DNA methylation on ASEs, especially in the context of cancer.

One of the most important determinants for effective splice-switching therapies was targeting AASEs with significant biological implications. To reveal the functional relevance of AASEs in LUAD, we performed deep analyses on AASEs-related genes. AASEs connected with many cancer hallmarks were enriched in “cell-substrate junction assembly” and “cell-matrix adhesion”. This result was consistent with previous studies suggesting that the interaction between cell and extracellular matrix is associated with multiple tumor-boosting pathways.^49,50^ Besides, our data showed that biological processes related to tumor proliferation and metastasis were primarily affected by AASEs. Thus, splice-switching therapeutics targeting AASEs associated with proliferation and metastasis is a promising approach for the treatment of LUAD.

We also noticed that one group of LUAD patients with a particular AASEs profile was immunogenic and predicted with a better response rate to immunotherapy. Previous studies aimed to subclass LUAD were only based on the expression profiles or somatic mutation status.^3,51^ Our study suggested that LUAD subtypes based on alternative splicing profiles may help identify candidates who are likely to benefit from immunotherapies. Besides, these results implied that splicing-switching therapeutics may reconstruct the tumor immune microenvironment and improve the response rate of LUAD patients to immunotherapy.

This study has some limitations. Although we applied multi-omics methods to systematically reveal the splicing dysregulation in LUAD based on large-scale and multicenter datasets, the specific function of ASEs and isoforms of many genes is still ambiguous. In-depth insights into the accurate biological function of each isoform would help to select prospective targets for splice-switching therapeutics. However, no high-throughput approaches are available to differentiate between therapeutic or pathogenic isoforms and isoforms with no role in tumor development. Thus, based on current technology, labor-intensive experiments are necessary to investigate the precise function of plentiful isoforms.

In summary, this study systematically investigated the landscape of AASEs in LUAD and unraveled the biological relevance of AASEs and their regulators from multiple perspectives. Besides, this study clarified the effects of splicing dysregulation on the tumor immune microenvironment and laid the foundation for designing splice-switching therapeutics for LUAD. The resource we provided in this study would promote the understanding of molecular characteristics of LUAD and facilitate basic research and precision medicine in LUAD.

## Materials and methods

### RNA sequencing data collection and curation

We collected two LUAD cohorts in this study. The first one is our integrated cohort, including dataset SRP074349, ERP001058, and Chinese LUAD patients we collected in the Cancer Hospital of the Chinese Academy of Medical Sciences. There are 108 LUAD samples in dataset SRP074349, 81 LUAD samples and 77 paired adjacent-normal samples in dataset ERP001058, and 112 LUAD samples and 76 paired adjacent-normal samples we collected, in which 48 LUAD samples and 46 normal samples have been published in our previous research.^52^ The other cohort is the TCGA LUAD cohort, including 514 tumor samples and 59 normal samples. All samples in these cohorts have undergone RNA sequencing. Raw RNA sequencing data in our integrated cohort were processed as follows. First, we employed FastQC to examine the quality of sequencing data and applied Trim-Galore to get rid of adapters and low-quality reads. Then, SpliceSeq^53^ was used to identify and quantify ASEs. Salmon^54^ was applied to calculate the expression level of genes and transcripts in each sample. For the TCGA LUAD cohort, we obtained alternative splicing profiles and gene expression profiles from TCGASpliceSeq^55^ and UCSC Xena^56^, respectively.

### Identification of high-confidence ASEs from high-quality samples

For our integrated cohort, we removed samples with a mapping rate <60% or detected ASEs <5000. As a result, 285 tumors and 145 paired adjacent-normal samples in this cohort were kept for further analysis, including 81 LUAD and 77 normal samples derived from dataset ERP001058, 108 LUAD samples from dataset SRP074349, and 96 LUAD and 68 normal samples we collected in China. Then we kept ASEs that were detected in at least 20% LUAD and 20% normal samples. For the TCGA LUAD cohort, we also kept ASEs detected in at least 20% LUAD and 20% normal samples. Those ASEs occurred in both cohorts were defined as high-confidence ASEs.

### Identifying and filtering AASEs in LUAD

In this study, percent spliced in (PSI), also known as the exon-inclusion ratio, was used to quantify alternative splicing events (ASEs). To explore aberrant ASEs (AASEs), we conducted Wilcoxon rank-sum test to measure the difference of each ASE between LUAD and adjacent-normal samples in our integrated dataset. We adjusted *p* values through the Benjamini–Hochberg method. ASEs were considered aberrant when satisfying the criteria of |ΔPSI| >0.1 (more than 10% difference in PSI value) and adjusted *p* values <0.05. Finally, 3688 AASEs were identified.

### Investigating the regulatory role of RBPs on AASEs

A total of 1779 RBPs were collected based on two previous reports.^57,58^ The R package “DEseq2”^59^ was applied to identify differentially expressed RBPs between LUAD and paired adjacent-normal samples. RBPs that met |logFc| > 1 and adjusted *p* values < 0.05 were considered to be differentially expressed. We then applied Spearman correlation analysis to test the relationship between the expression levels of 100 differentially expressed RBPs and the PSI values of 3688 AASEs. Criteria for |r| > 0.5 (absolute value of the correlation coefficients larger than 0.5) and adjusted *p* value < 0.05 indicated that differentially expressed RBPs would play a regulatory role on their corresponding AASEs. To promote the robustness of the RBP-ASE pairs identified, we applied DeepBind (version 0.11)^60^ to predict whether the RBP could bind to ±300 bp around the splice sites of its corresponding ASEs. Due to the limited training sets, DeepBind can only predict the binding region of 85 human RBPs, containing three differentially expressed RBPs (IGF2BP2, KHDRBS2, and YBX2) in this study. For each RBP, chromosome regions with scores greater than the first quantile of all regions around splicing sites of AASEs were recognized as the candidate binding sites of this RBP. We integrated this binding information with the significantly correlated RBP-ASE pairs and revealed more solid interactions between RBPs and ASEs. Cytoscape^61^ was employed to build RBPs-ASEs networks based on the identified interactions. To further scan the position of motifs for the three differentially RBPs, we applied rMAPS2^62^ running for ES events with a sliding window of 50 bases.

### Investigating cis-sQTLs and trans-sQTLs in LUAD

To make the sQTLs we identified robustly, we combined 506 TCGA LUAD samples with 57 LUAD samples we collected in China with mutation data. ASEs detected in at least 70% of LUAD samples and SNVs observed in more than two LUAD samples were retained for further analysis. The commonly-used R package “matrixEQTL” was applied to identify sQTLs.^63^ In detail, the additive linear regression model was used to evaluate the effects of somatic mutations on ASEs. We included gender, background, batch, age, and TNM stage as covariates to reduce their effects on the sQTLs results. We investigated local (cis) and distant (trans) sQTLs separately. Cis-sQTL is within 100 Kbp around its’ corresponding splicing site. The location of splicing sites (hg19) was obtained from the TCGASpliceseq database. SNV-ASE pairs were kept when the ASE can be detected in more than two corresponding mutant samples. FDR <0.01 suggested that the SNV-ASE pairs were significant and this SNV was an sQTL.

### Revealing the regulatory pattern of DNA methylation on ASEs

DNA methylation data generated by Illumina Human Methylation 450 K BeadChip were obtained from UCSC Xena. Only CpG sites within the boundary of differentially spliced exons were reserved for differential testing. The boundary was defined as 39 nucleotides in exonic regions and 200 nucleotides in intronic regions around splice sites according to Castle’s study.^27^ Differentially methylated CpG sites were detected by R package “CHAMP”^64^ according to the criteria of adjusted *p* values < 0.05 and |Δmeth| > 0.15 (more than 15% difference in methylation). Subsequently, we correlated these differential CpG sites with their corresponding AASEs. To investigate the regulatory role of DNA methylation on multiple cancer types, we collected 30 cancer types’ DNA methylation data from UCSC Xena and alternative splicing profiles from TCGASpliceSeq. CpG sites at the boundaries of alternatively spliced exons were kept and investigated their correlation with corresponded ASEs. Adjusted *p* value < 0.05 and |r| > 0.3 suggested the CpG-ASE pairs were significantly correlated.

### Investigating tumor immune microenvironment of LUAD samples

We used CIBERSORT^65^ to estimate the infiltration level of 22 immune cell types and then employed TIMER2.0^66^ to confirm the immune cell signatures in LUAD samples. The ssGSEA analysis was conducted to evaluate the activation degree of 17 immune pathways defined by ImmPort.^67^ To further investigate the immune microenvironment of LUAD, we measured the expression levels of 78 immunomodulators obtained from one previous report.^34^

### Comparing tumor immune microenvironment among three LUAD subtypes

We classified LUAD samples into three subtypes according to AASEs profiles through the R package “ConsensusClusterPlus”^68^ and compared the immune signatures (immune pathways, immune cell abundance, and immunomodulators) among these subtypes. Analysis of variance and *t*-test were applied to investigate the difference in immune pathways among the three subtypes due to the normal distribution of these values. The Kruskal–Wallis test and Wilcoxon rank-sum test were used to evaluate the difference in the level of immune infiltration and immunomodulators among the three subtypes. To predict the clinical responses to immune checkpoint blockades among LUAD patients, we utilized the TIDE algorithm^69^ with setting “NSCLC” in the parameter “Cancer type” and “No” in the parameter of “Previous immunotherapy.” The Chi-square test was used to verify the relevance between the three clusters and the immunotherapy response.

### Immunohistochemical (IHC) staining and scoring

Immunohistochemical (IHC) staining and scoring of CD8 were the same as in our previous research.^52^ In detail, we spliced formalin-fixed, paraffin-embedded tumor tissues into 4 mm slides for IHC staining with CD8 antibody (1:50, ZSGB Bio, catalog No: ZA-0508) using an automated Leica Bond staining system according to the manufacturer’s protocol. The PD-L1 IHC staining procedure was performed with the PD-L1 IHC 22C3 pharmDx (Dako, Inc.) companion diagnostic test on the Dako Autostainer Link 48 platform. For scoring the IHC image, Histoscore (H-score) was calculated by multiplying the proportion of positive cells in the sample (0–100%) by the average intensity of the positive staining (1+, 2+, or 3+) to obtain a score ranging between 0 and 300 as previously described.^70^

## Supplementary information

Supplementary Materials

Supplementary Tables

## Data Availability

Raw sequencing data originally generated in this study are available under Accession No. HRA001814 in the Genome Sequence Archive for Human of Beijing Institute of Genomics, Chinese Academy of Sciences.
