# Supplementary material for: Multi-omics analysis reveals RNA splicing alterations and their biological and clinical implications in lung adenocarcinoma
Source: Signal Transduct Target Ther. 2022 Aug 22;7:270. doi: 10.1038/s41392-022-01098-5 (PMC9393167; doi:10.1038/s41392-022-01098-5)
Supplement: Supplementary file 1 — Supplementary Materials [file 41392_2022_1098_MOESM1_ESM.docx]

Supplementary Materials for

Multi-omics analysis reveals RNA splicing alterations and their biological and clinical implications in lung adenocarcinoma

Quanyou Wu^1,#^, Lin Feng^1,#^, Yaru Wang^1,#^, Yousheng Mao^2^, Xuebing Di^1^, Kaitai Zhang^1,^*, Shujun Cheng^1,^*, Ting Xiao^1,^*

Correspondence to: [xiaot@cicams.ac.cn](mailto:xiaot@cicams.ac.cn); [chengshj@cae.cn](mailto:chengshj@cae.cn); [zhangkt@cicams.ac.cn](mailto:zhangkt@cicams.ac.cn)

**This PDF file includes:**

Materials and Methods

Figures. S1 to S8

Tables S1 to S13 (See attached Excel files)

**Materials and methods**

**Specimen collection**

The collection procedure of LUAD and adjacent-normal samples in this study is similar to our previous research.^1^ In detail, samples used for this study were collected from National Cancer Center and Cancer Hospital of Chinese Academy of Medical Sciences & Peking Union Medical College. Patients who were not treated with any anti-cancer therapies before surgery were randomly selected. Primary tumor tissues and paired adjacent-normal tissues (> 3 cm apart from tumor edge) were surgically resected and transferred to sterile cryovials. The tumor tissues and adjacent-normal tissues stained with hematoxylin and eosin were evaluated by two pathologists to identify the tumor purity. Tumor tissues exhibiting >50% tumor cells and the adjacent-normal tissues containing no tumor cells were kept for RNA extraction. All collected tissues were processed with RNAlater Stabilization Solution (Invitrogen, catalog No: AM7021, Carlsbad, CA) and stored at -80°C. This study was approved by the Research Ethics Committee of National Cancer Center and Cancer Hospital of Chinese Academy of Medical Sciences & Peking Union Medical College. All patients provided informed consent for the use of their tumor specimens.

**RNA extraction and library preparation**

RNA was extracted from tissues using TRIzol reagent kit (Ambion, Invitrogen, USA) according to the reagent protocols. The RNA integrity and concentration were measured through an Agilent 2100 Bioanalyzer (Agilent, CA, USA) and a NanoDrop ND-1000 spectrophotometer (NanoDrop Technologies, Wilmington, USA). RNA samples exhibiting an RNA integrity number (RIN) greater than 6.0 were kept for further use. A total amount of 2 mg RNA per sample was used as the input material for the library preparations. Sequencing libraries were generated using NEBNext® UltraTM RNA Library Prep Kit for Illumina® (#E7530L, NEB, USA) and the libraries were sequenced on an Illumina platform (2 X 150-bp paired-end read length).

**Identification of drug-related RBPs**

We first retrieved the sensitivity information for all tested drugs across LUAD cell lines from Genomics of Drug Sensitivity in Cancer (GDSC).^2^ GDSC maintained two datasets, GDSC1 and GDSC2. If GDSC2 deposits the IC50 value for one drug, then we considered this value as the measurement of drug sensitivity, else, we used the IC50 value in GDSC1. We conducted Spearman correlation analysis between the expression levels of top 20 RBPs with the highest regulatory capabilities on AASEs and the IC50 values of drugs across 12 LUAD cell lines. The drug-RBP pairs with p-value <0.05 and |r| >0.5 were considered significant and visualized by Cytoscape.

**Investigation of the functional implications of AASEs in LUAD**

We employed the functional diversity analysis module of TappAS to interrogate the varying features of different isoforms belonging to the same AASEs-related gene.^3^ GO enrichment analysis was further performed for AASEs-related genes through the R package “clusterProfiler”.^4^ We collected 14 cancer hallmark gene sets from one previous report^5^ and evaluated the activation degree of these hallmarks through the ssGSEA method provided by the R package “GSVA”.^6^ The relationship between AASEs and the degree of these hallmarks was measured by Spearman correlation analysis. Hive plot was applied to represent the relationship among AASEs, cancer hallmarks, and the GO terms.^7^

**Characterizing the biological features of the three AASE subtypes**

Pathway enrichment analysis was performed to investigate the biological characteristics of the three AASE subtypes. In detail, we firstly collected pathway annotation from the Reactome (using R package reactome.db v1.70.0), KEGG (using the R package KEGGREST v1.28.0) databases, and hallmark gene sets from MSigDB (v7.5.1) (<https://www.gsea-msigdb.org/gsea/msigdb/>). We joined these annotations and performed gene set variation analyses (using the R package GSVA 1.36.3) to obtain single-sample enrichment scores for each pathway. To identify subtype-specific pathways, we conducted Wilcoxon rank-sum test using the pathway enrichment scores between samples in each subtype versus samples in other subtypes. Multiple testing was adjusted by the Benjamini-Hochberg method.

**Calculation of telomerase activity and stemness index**

The telomerase activity of each LUAD sample was calculated by the EXTEND algorithm,^8^ which robustly estimated telomerase enzymatic activity from the expression of a 13-gene signature. Stemness indices were measured by the one-class logistic regression machine-learning algorithm as previously described, which accurately measured the stemness degree of each sample based on transcriptome and epigenetic features.^9^

**Finding potential drugs for the treatment of three LUAD subtypes**

Connectivity Map (Cmap),^10^ a public tool that enables researchers to identify compounds that may inhibit diseases based on gene expression profiles, was employed to predict which drugs might be effective for the treatment of each LUAD subtype. We compared the expression profiles of each LUAD subtype with adjacent-normal samples and selected the top 300 genes (150 upregulated and 150 downregulated) for Cmap analysis. Specific analysis based on Cmap tools was conducted to further interrogate the action mechanisms pattern of drugs that most likely inhibit LUAD.

**Cell lines and reagents**

Human LUAD cell lines A549, NCI-H2347, and NCI-H441 were purchased from the American Type Culture Collection (ATCC, Manassas, USA) and cultured in RPMI-1640 (Gibco, Grand Island, NY, USA) medium supplemented with 10% FBS (Gibco, New York, USA) at 37°C with 5% CO_2_. All cell lines were authenticated by short tandem repeat DNA proﬁling (Microread Gene Technology, Beijing, China). AT13387 (Selleck, Beijing, China) and nomilin (Selleck, Beijing, China) were added to LUAD cell lines at the appropriate concentrations.

**Western blot assay and antibodies**

Fifteen paired LUAD tumor tissues and adjacent normal tissues stored in a -80°C refrigerator were thawed, and then cut into pieces for extracting the proteins. For the LUAD cell lines A549, NCI-H2347, and NCI-H441, trypsin was used for cell digestion, and cells were centrifuged for extracting the proteins. The RIPA lysis buffer (Applygen, China) supplemented with PMSF (Sigma, Missouri, America), and Protease Inhibitor Cocktail (Thermo Fisher, Massachusetts, America) was used to lysed tissues and cells on ice, then proteins were quantiﬁed using the BCA Protein Assay Kit (Thermo Fisher, Massachusetts, America). Equal amounts of the cell protein lysates (30 mg) were separated by 10% SDS-PAGE and transferred to a polyvinylidene diﬂuoride (PVDF) membrane (Millipore, Darmstadt, Germany). After being blocked with 5% nonfat dry milk in 1X phosphate-buffered saline (PBS) and 0.1% Tween 20 solution (Solarbio, Beijing, China), membranes were incubated overnight at 4°C with the following primary antibodies: HSP90AB1 (ABGENT, China, 1:1000), NUMB (Cell Signaling Technology, USA, 1:1000) and GAPDH (Proteintech, China, 1:10000). The membranes were then incubated with the HRP-conjugated Affinipure Goat Anti-Rabbit IgG (Proteintech, China, 1:10000) for 1 hour at room temperature. The speciﬁc bands were detected using an ECL detection kit (Applygen, China) and captured on an ImageQuant LAS 4010 system (GE Healthcare, NJ, USA).

**Cell proliferation assays**

A Cell Counting Kit-8 (CCK8, DOJINDO, Japan) assay was conducted according to the manufacturer's procedure. In detail, the cultured cells (A549, NCI-H2347, NCI-H441) were harvested, counted (5,000 cells/well) and, cultured in 96-well plates at 37°C with 5% CO_2_. The cells were cultured for 4 hours to adhere to the cell wall, and then the culture medium with different drug concentrations was changed. Then, CCK8 solution was added to each well, and each well was measured spectrophotometrically at 450 nm after incubating for 2 hours. We consider this to be hour “0” of the proliferation curve. Then, every 24 hours, cell growth was detected in the same manner. We detected cell proliferation for 96 hours after hour 0. For the concentrations, we used AT13387 (0 nM, 250 nM, 500 nM, 750 nM, 1000 nM) and nomilin (0 uM, 100 uM, 200 uM, 300 uM, 400 uM) to determine the drug concentration. and use AT13387 (1000 nM) and nomilin (400 uM) to conduct formal experiments.

**Colony formation assay**

In the 6-well plates, 1000 cells (A549, NCI-H2347, NCI-H441) per dish were inoculated at 37°C with 5% CO_2_. The cells were cultured for 4 hours to adhere to the cell wall. Then, we added the corresponding concentration (AT13387: 1000 nM, nomilin: 400 um) of drugs to the cell culture medium, observed the formation of cell clones, stopped cell culture when the clone size was appropriate (7-14 days), and proceed to the staining step. After washing with 4 °C precooled 1X PBS (Gibco, Grand Island, NY, USA), cultures were fixed with 4 °C pre-chilled methanol (Sangon, ShangHai, China) for 10 min, then stained with 0.5% crystal violet for 10 min. Imaging and cell clone counting were performed with the GBOX-F3EE (SYNGENE, Cambridge, UK). Each experiment was made in triplicate.

**Statistics**

All figure plotting and statistical analyses in this study were performed using R software. Statistical tests were two-sided and p-values less than 0.05 were considered as statistical significance unless indicated otherwise.

**References**

1 Xu, J. Y. *et al.* Integrative Proteomic Characterization of Human Lung Adenocarcinoma. *Cell* **182**, 245-261.e217 (2020).

2 Yang, W. *et al.* Genomics of Drug Sensitivity in Cancer (GDSC): a resource for therapeutic biomarker discovery in cancer cells. *Nucleic Acids Res.* **41**, D955-961 (2013).

3 de la Fuente, L. *et al.* tappAS: a comprehensive computational framework for the analysis of the functional impact of differential splicing. *Genome Biol.* **21**, 119 (2020).

4 Yu, G., Wang, L. G., Han, Y. & He, Q. Y. clusterProfiler: an R package for comparing biological themes among gene clusters. *Omics* **16**, 284-287 (2012).

5 Uhlen, M. *et al.* A pathology atlas of the human cancer transcriptome. *Science* **357**, 660-+ (2017).

6 Hänzelmann, S., Castelo, R. & Guinney, J. GSVA: gene set variation analysis for microarray and RNA-seq data. *BMC Bioinformatics* **14**, 7 (2013).

7 Krzywinski, M., Birol, I., Jones, S. J. & Marra, M. A. Hive plots--rational approach to visualizing networks. *Brief. Bioinform.* **13**, 627-644 (2012).

8 Noureen, N. *et al.* Integrated analysis of telomerase enzymatic activity unravels an association with cancer stemness and proliferation. *Nat. Commun.* **12**, 139 (2021).

9 Malta, T. M. *et al.* Machine Learning Identifies Stemness Features Associated with Oncogenic Dedifferentiation. *Cell* **173**, 338-354.e315 (2018).

10 Subramanian, A. *et al.* A Next Generation Connectivity Map: L1000 Platform and the First 1,000,000 Profiles. *Cell* **171**, 1437-1452.e1417 (2017).

**Figure. S1.**


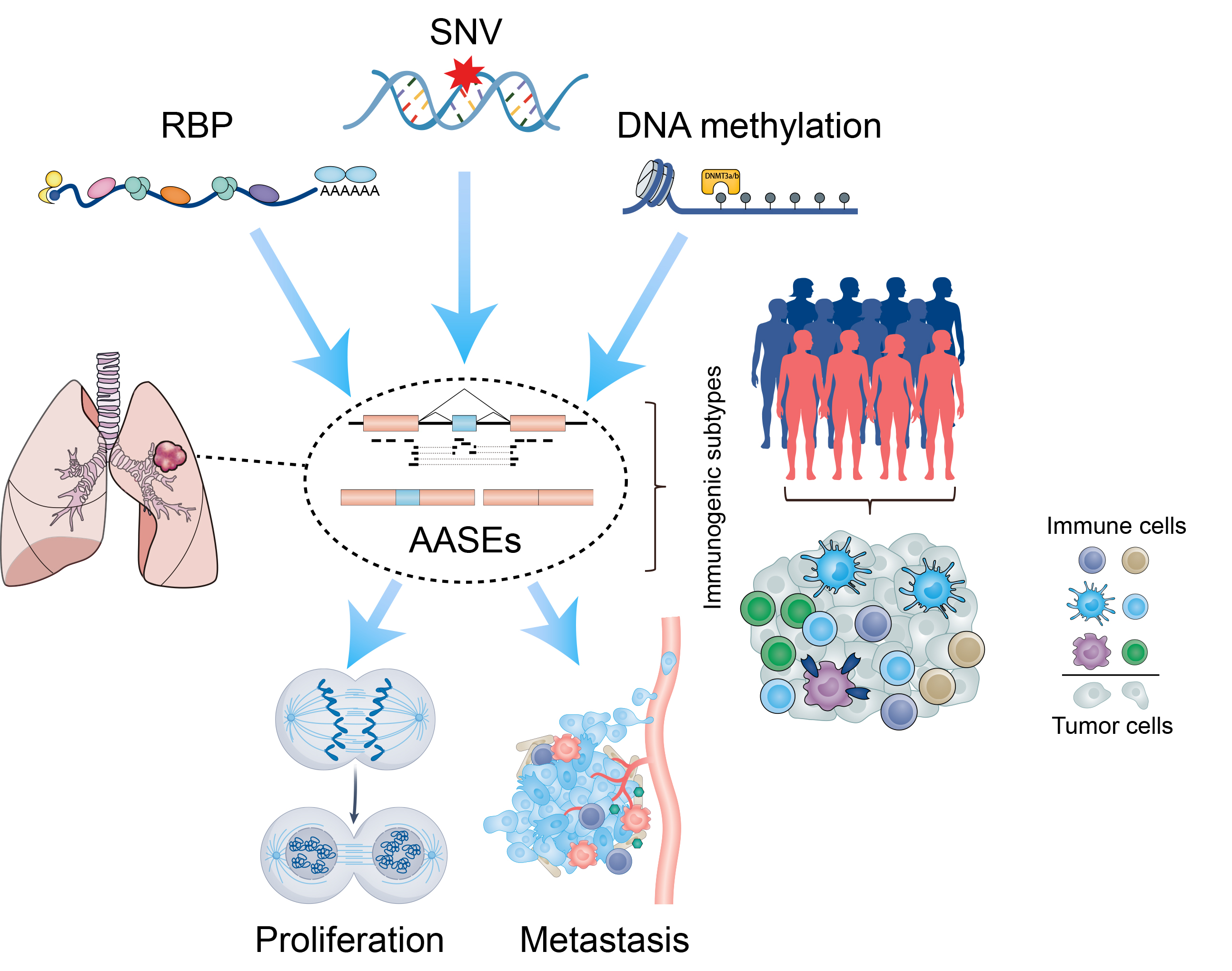


**Figure S1 | Graphical abstract of this study.** In this study, we first detected AASEs in LUAD samples. Then we unraveled the upstream regulators of AASEs from the perspective of RBP, SNV, and DNA methylation. These AASEs in LUAD mainly affected pathways related to tumor proliferation and metastasis. Based on AASEs profiles, we identified one immunogenic LUAD subtype with a better prognosis and a higher response rate to immunotherapy.

**Figure. S2**
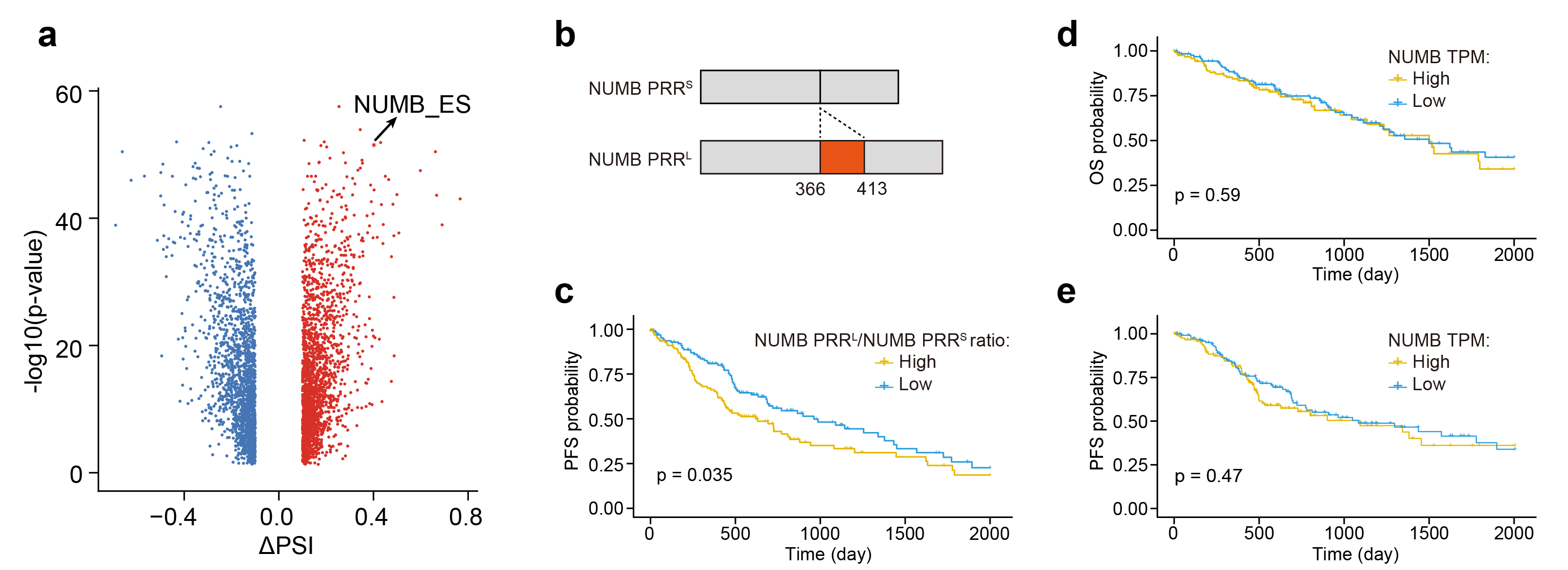


**Figure S2 | The significance of alternative splicing of NUMB in LUAD** (a) Volcano plot showing that the exon skipping event of NUMB was one of the most significant AASEs in LUAD. (b) Schematic diagram showing that NUMB PRR^L^ has additional 48 amino acids (marked in red) compared to NUMB PRR^S^. (c-e) Kaplan-Meier survival curves comparing the progression-free survival (PFS) of high and low NUMB PRR^L^/NUMB PRR^S^ ratio subgroups (c); the OS (d) and PFS (e) of high and low NUMB expression subgroups. Patients were stratified into high (top 25th percentile) and low (bottom 25th percentile) subgroups based on their PSI value or total Numb expression level.

**Figure. S3.**

**
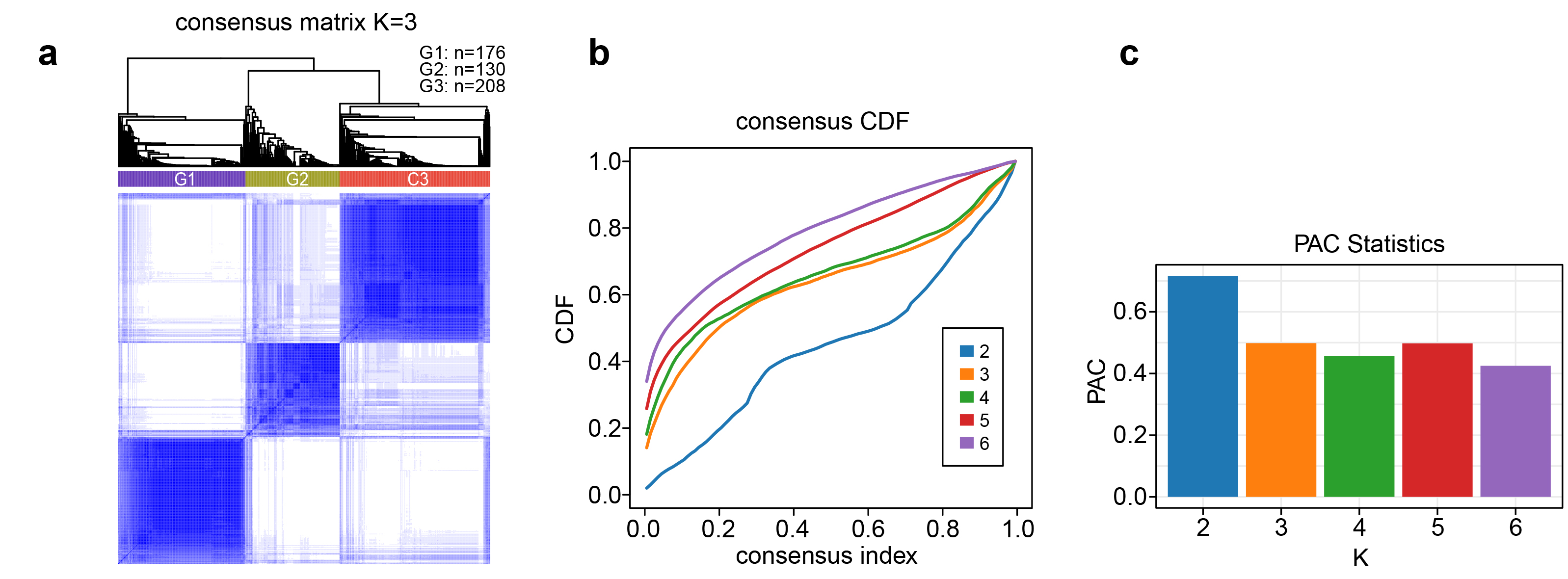
**

**Figure S3 | Consensus clustering analysis calculated on all AASEs.** (a) Heatmap of the consensus matrix based on all AASEs for cluster number k = 3 where consensus values ranging from 0 (samples never clustered together) to 1 (samples always clustered together) are marked by white to dark blue. (b) The cumulative distribution function (CDF) curve of the consensus matrix for each cluster number k (from 2 to 6). (c) Proportion of ambiguous clustering (PAC) statistics for each k.

**Figure. S4.**


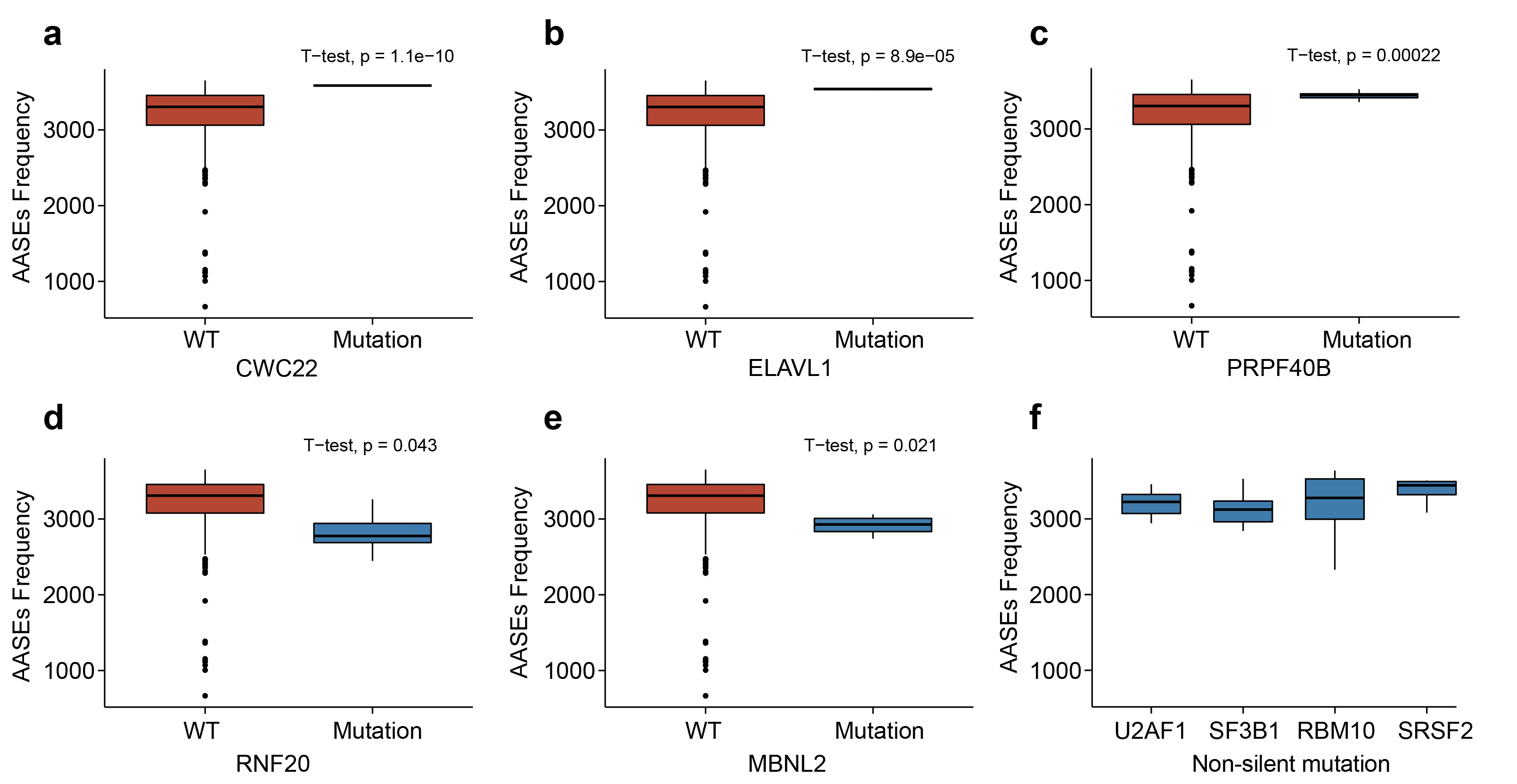


**Figure S4 | The correlation between AASEs frequency and gene alterations.** (a-e) Box plot showing the effects of non-silent mutations of CWC22 (a), ELAVL1 (b), PRPF40B (c), RNF20 (d), and MBNL2 (e) on the AASEs frequency. (f) The AASEs frequency of LUAD samples having U2AF1, or SF3B1, or RBM10, or SRSF2 non-silent mutations.

**Figure. S5.**
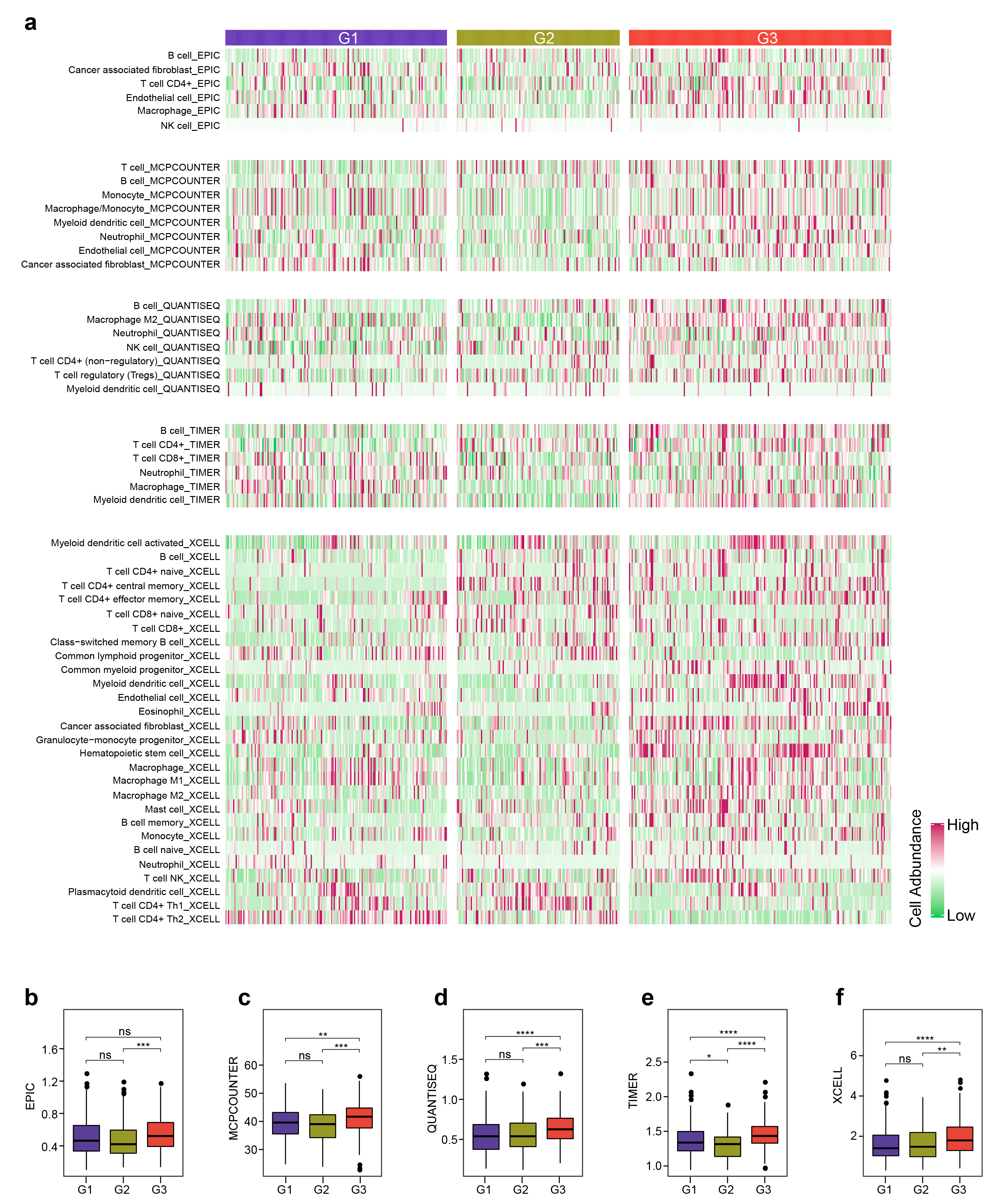


**Figure S5 | Immune cell infiltration levels measured by TIMER2.0.** (a) The infiltration level of immune cells that exhibited significant differences among the three LUAD subtypes, calculated by EPIC, MCP-counter, quanTIseq, TIMER, and xCell algorithms respectively. (b-f) Box plots showing the infiltration level of total immune cells in the three LUAD subtypes, calculated by EPIC (b), MCP-counter (c), quanTIseq (d), TIMER (e), and xCell (f) respectively.

**Figure. S6.**


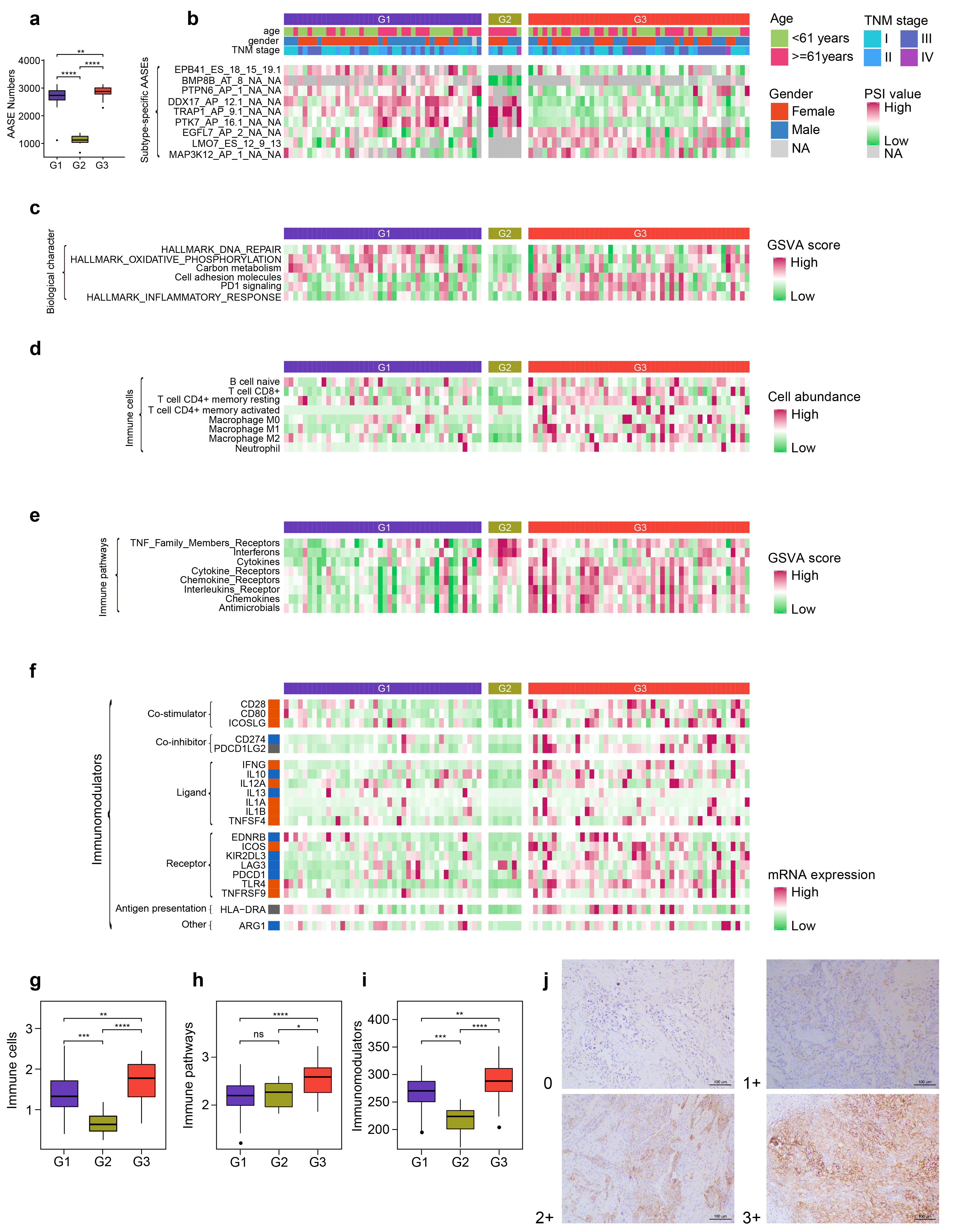


**Figure S6 | Characteristics of AASEs subtypes identified in our own cohort.** (a) Box plot showing the AASEs frequency of LUAD samples in each subtype. (b) Heatmap representing the subtype-specific AASEs in each LUAD subtype. (c) The biological characteristics of each LUAD subtype. (d-f) Heatmap showing the infiltration level of immune cells (d), the activation degree of immune pathways (e), and the expression of immunomodulators (f) that are significantly different among the three LUAD subtypes. (g-i) Box plots exhibiting the infiltration level of total immune cells (g), the activation degree of total immune pathways (h), and the expression of total immunomodulators (i) in the three LUAD subtypes. (j) Representative IHC images showing each intensity level of PD-L1 expression.

**Figure. S7.**


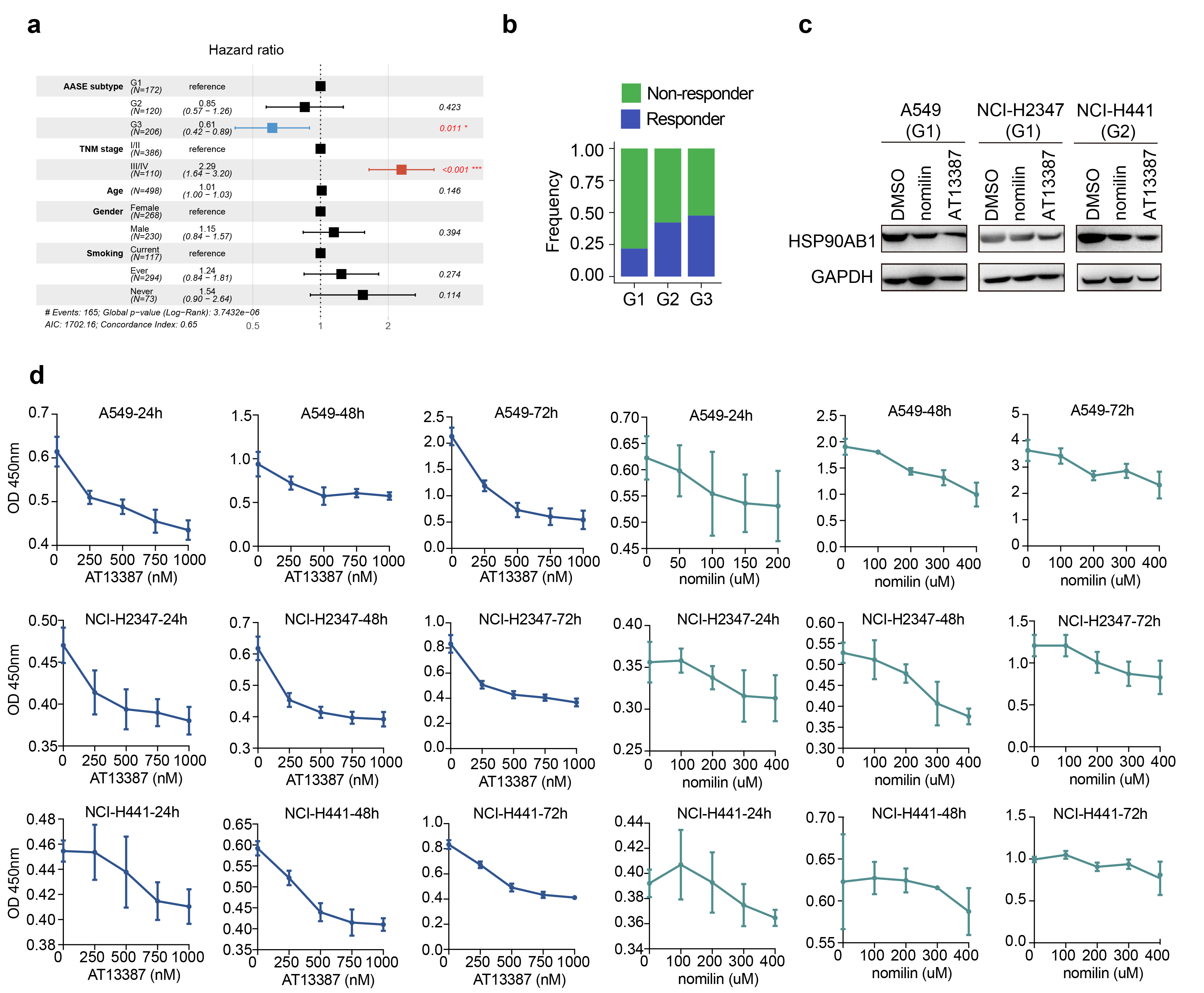


**Figure S7 | The clinical implications of AASEs subtypes of LUAD.** (a) Forest plot based on hazard ratios of a multivariate Cox regression analysis for the overall survival of patients in the TCGA LUAD cohort. Covariates used in the Cox regression are shown in the leftmost column. The second column lists all modeled levels of the covariates, with the top-most level being the reference level for each covariate. HR estimates with 95% confidence intervals relative to these references are shown in the third column and visualized as boxes and horizontal lines, respectively, in the fourth column. The dotted vertical line marks an HR of 1. P-values indicating statistical significance are shown on the right. (b) The response rate to immunotherapies of the three AASEs LUAD subtypes. (c) Western blotting analysis of HSP90AB1 in the three LUAD cell lines. Each cell line was treated with DMSO, nomilin, and AT13387, separately. (d) Viabilities of three LUAD cell lines treated with AT13387 and nomilin at concentrations as indicated for 24h, 48h, and 72h. Representative data from six biological repeats were shown (mean ± SD).

**Figure. S8.**


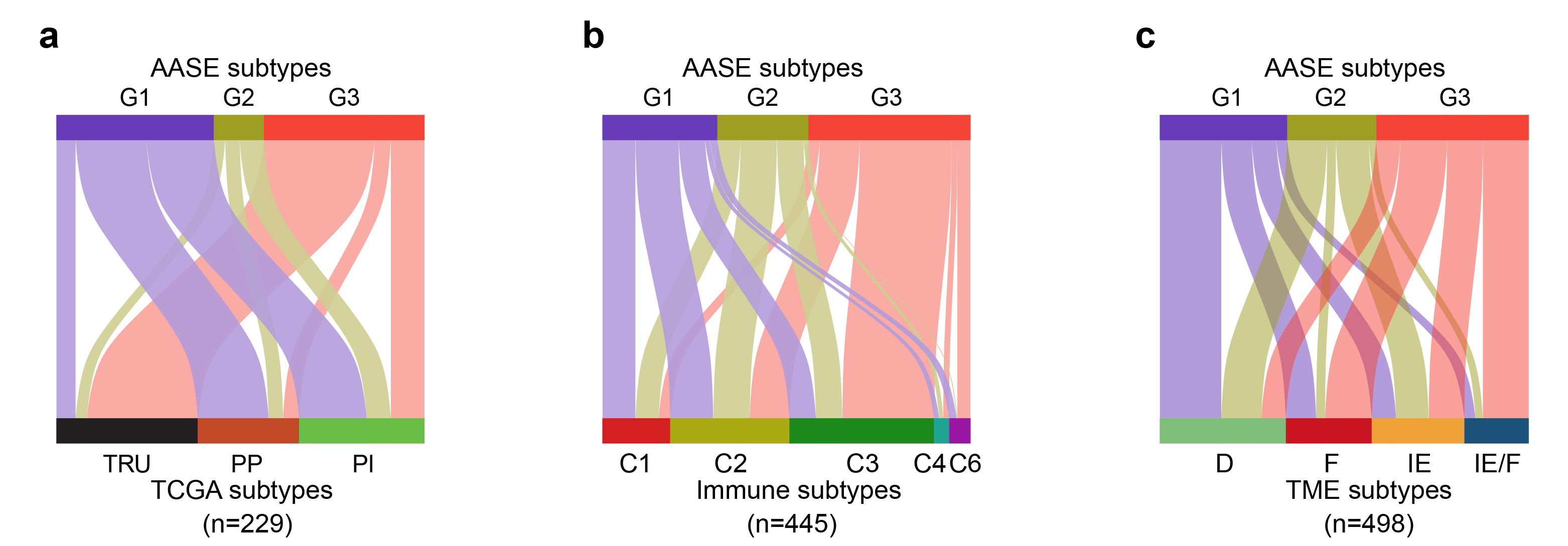


**Figure S8 | The association between AASEs subtypes and known subgroups defined by other researchers.** (a-c) Comparison between the three AASEs subtypes and the TCGA subtypes (a), the immune subtypes (b), and the TME subtypes (c).

**Table S1. All aberrant alternative splicing events (AASEs) identified in LUAD**

**Table S2. Differentially expressed RNA binding proteins (RBPs) in LUAD**

**Table S3. RBP-ASE pairs**

**Table S4. The result of gene ontology enrichment analysis based on RBP-regulated AASEs**

**Table S5.** **More reliable RBP-ASE pairs**

**Table S6. Significant correlation between AASEs and cancer hallmarks**

**Table S7.** **Classification of TCGA LUAD sample based on AASE profiles**

**Table S8.** **Subtype-specific AASEs**

**Table S9. Infiltration levels of 22 immune cell types in TCGA LUAD cohort**

**Table S10.** **Activation degree of 17 immune pathways in TCGA LUAD cohort**

**Table S11.** **Classification of our own LUAD sample based on AASE profiles**

**Table S12.** **Experimentally validating the immune characteristics of each subtype**

**Table S13.** **The subtype of 55 LUAD cell lines**
